# TME/NIR Dual‐Responsive Zinc‐Based Targeted Nanoagonist for Multimodal Amplification of STING‐Mediated Cancer Immunotherapy

**DOI:** 10.1002/advs.202523182

**Published:** 2026-06-09

**Authors:** Kepeng Hu, Jiawei Wang, Wei Zhang, Zhixuan Wu, Jing Chen, Yujing He, Xiyue Duan, Yaoting He, Xin Xin, Haoyi Xiang, Yijing Wang, Chengwei Wu, Yang Yi, Qing Meng, Yuqian Qiao, Kui Cheng, Xiaojun Long, Zhangfa Song

**Affiliations:** ^1^ Department of Colorectal Surgery Sir Run Run Shaw Hospital School of Medicine Zhejiang University Hangzhou Zhejiang China; ^2^ Zhejiang Key Laboratory of Biological Treatment Hangzhou Zhejiang China; ^3^ Key Laboratory of Integrated Traditional Chinese and Western Medicine Research on Anorectal Diseases of Zhejiang Province Hangzhou Zhejiang China; ^4^ School of Basic Medical Sciences and Forensic Medicine Hangzhou Medical College Hangzhou Zhejiang China; ^5^ School of Materials Science and Engineering State Key Laboratory of Silicon and Advanced Semiconductor Materials Zhejiang University Hangzhou Zhejiang China; ^6^ School of Materials Science and Engineering Nankai University Tianjin China

**Keywords:** cancer immunotherapy, cGAS‐STING pathway, immunogenic cell death, nano‐metabolite, tumor microenvironment response

## Abstract

The precise activation of the cGAS‐STING pathway for effective tumor immunotherapy remains a significant challenge due to the complexity of immune responses and tumor microenvironment (TME) limitations. Here, a multifunctional nanoagonist, cRGD‐PDA/ZIF8@ICG/TPT (cDZ@IP), was developed to realize nano‐metabolite–driven multimodal synergistic activation of the STING pathway and enhanced immune recognition. The agonist combines cRGD peptide‐targeted polydopamine‐coated zeolitic imidazolate framework‐8 with the photosensitizer indocyanine green and the chemotherapeutic drug topotecan. Upon near‐infrared laser irradiation, cDZ@IP degrades within the TME, generating high levels of reactive oxygen species, inducing mitochondrial stress, and releasing endogenous mitochondrial DNA. Additionally, topotecan enhances DNA damage accumulation by inhibiting nuclear DNA repair. Zn^2^^+^ released from the agonist further amplifies cGAS‐STING pathway activation, thereby ensuring a robust immune response. The mild photothermal therapy‐induced immunogenic cell death promotes the initiation of antitumor immunity, while also enhancing the effectiveness of immune checkpoint blockade. In vivo studies show that cDZ@IP significantly inhibits primary and distant tumor growth and prevents lung metastasis, providing a promising strategy for STING pathway‐targeted cancer immunotherapy.

## Graphical abstract

A multifunctional nanoagonist (cDZ@IP) enables nano‐metabolite–driven multimodal activation of the STING pathway and enhanced immune recognition, achieving potent antitumor immunity and suppressing tumor growth and metastasis. This strategy highlights the rational design of therapeutic metabolites and establishes a new paradigm bridging nanomedicine and cancer immunotherapy.

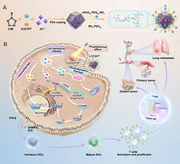

## 1 Introduction

The precise activation of the cyclic GMP‐AMP synthase‐stimulator of interferon genes (cGAS‐STING) pathway remains a major challenge in tumor immunotherapy [1, 2, 3], primarily due to the difficulty in achieving controlled and specific activation in the complex tumor microenvironment (TME) [4, 5, 6]. The cGAS‐STING pathway plays a pivotal role in recognizing cytosolic DNA, which serves as a danger signal for the innate immune system [7, 8, 9, 10]. The DNA sensor cGAS detects both exogenous and endogenous DNA, synthesizing cyclic GMP‐AMP (cGAMP) to activate STING and ultimately promote the secretion of type I interferons (IFN‐I), cytokines, and chemokines, key processes in antitumor immunity [11, 12, 13, 14, 15, 16]. Despite this potential, the clinical application of traditional STING agonists has been hindered by issues of poor stability, limited targeting, and systemic toxicity [17, 18, 19, 20], necessitating the development of more effective and targeted strategies for precise STING pathway activation. Recent advances in nanomedicine have provided new opportunities to activate the cGAS‐STING pathway by promoting cytosolic DNA accumulation or delivering STING agonists, thereby enhancing innate immune activation and facilitating the transformation of immunologically “cold” tumors into “hot” tumors [21].

In addition, accumulating evidence suggests that mitochondrial and nuclear DNA, released during chemotherapy, radiotherapy, or other forms of treatment, can act as triggers for cGAS‐STING activation [22, 23, 24, 25, 26, 27]. Various DNA‐damaging chemotherapeutic agents, such as anthracyclines [28], platinum‐based drugs [29], and topoisomerase inhibitors [30, 31], have been reported to promote STING activation through cytosolic DNA accumulation or micronucleus formation. Among them, topotecan (TPT), a clinically approved topoisomerase I inhibitor, is capable of inducing replication‐associated DNA damage and enhancing cytosolic DNA release, thereby facilitating immune activation. However, the clinical efficacy of such approaches is often limited by high toxicity, off‐target effects, and inadequate immune activation [32, 33, 34]. To overcome these challenges, strategies involving controlled‐release STING agonists, such as those used in photodynamic therapy (PDT), have been explored. For instance, indocyanine green (ICG) generates reactive oxygen species (ROS) under near‐infrared (NIR) irradiation, inducing mitochondrial damage and releasing mitochondrial DNA (mtDNA) to activate the cGAS‐STING pathway [35, 36]. However, critical challenges remain in enhancing cGAS sensitivity to DNA binding, boosting cGAMP production, and improving the affinity between cGAMP and STING [17, 37]. Furthermore, external physical stimuli such as radiotherapy or ultrasound have been reported to induce cytosolic DNA release and transient activation of the cGAS‐STING pathway [5]. However, such activation is often insufficient and short‐lived due to the immunosuppressive tumor microenvironment, thereby necessitating synergistic strategies to achieve sustained immune activation. In this context, nanoparticle‐based systems offer a promising approach to amplify and prolong STING signaling through integrated endogenous and exogenous stimuli [38].

Another promising strategy involves the use of zinc ions (Zn^2^^+^), which have been shown to enhance the cGAS‐STING signaling pathway, potentially improving the efficacy of STING‐based cancer immunotherapies [39]. Similarly, recent studies have demonstrated that metal ion‐assisted nanoplatforms can enhance cGAS sensitivity to cytosolic DNA and amplify STING signaling through synergistic ROS generation and mitochondrial damage [40]. In addition, chemotherapy‐induced immunogenic cell death (ICD) can trigger the release of tumor‐associated antigens, contributing to antitumor immunity [41, 42]. However, chemotherapy agents such as doxorubicin and oxaliplatin often fail to sufficiently induce ICD due to non‐specific biodistribution, rapid clearance, and insufficient tumor cell death [43, 44]. To overcome these limitations, mild photothermal therapy (mPTT) can be employed as an adjunct to chemotherapy [45, 46, 47]. mPTT enhances ICD by providing an exogenous trigger for the release of tumor‐associated antigens, thus improving the overall immunogenic response and further boosting antitumor immunity [48, 49, 50, 51]. This synergistic approach addresses the deficiency in chemotherapy‐induced ICD and optimizes the therapeutic potential of STING‐based cancer immunotherapy.

**SCHEME 1:**
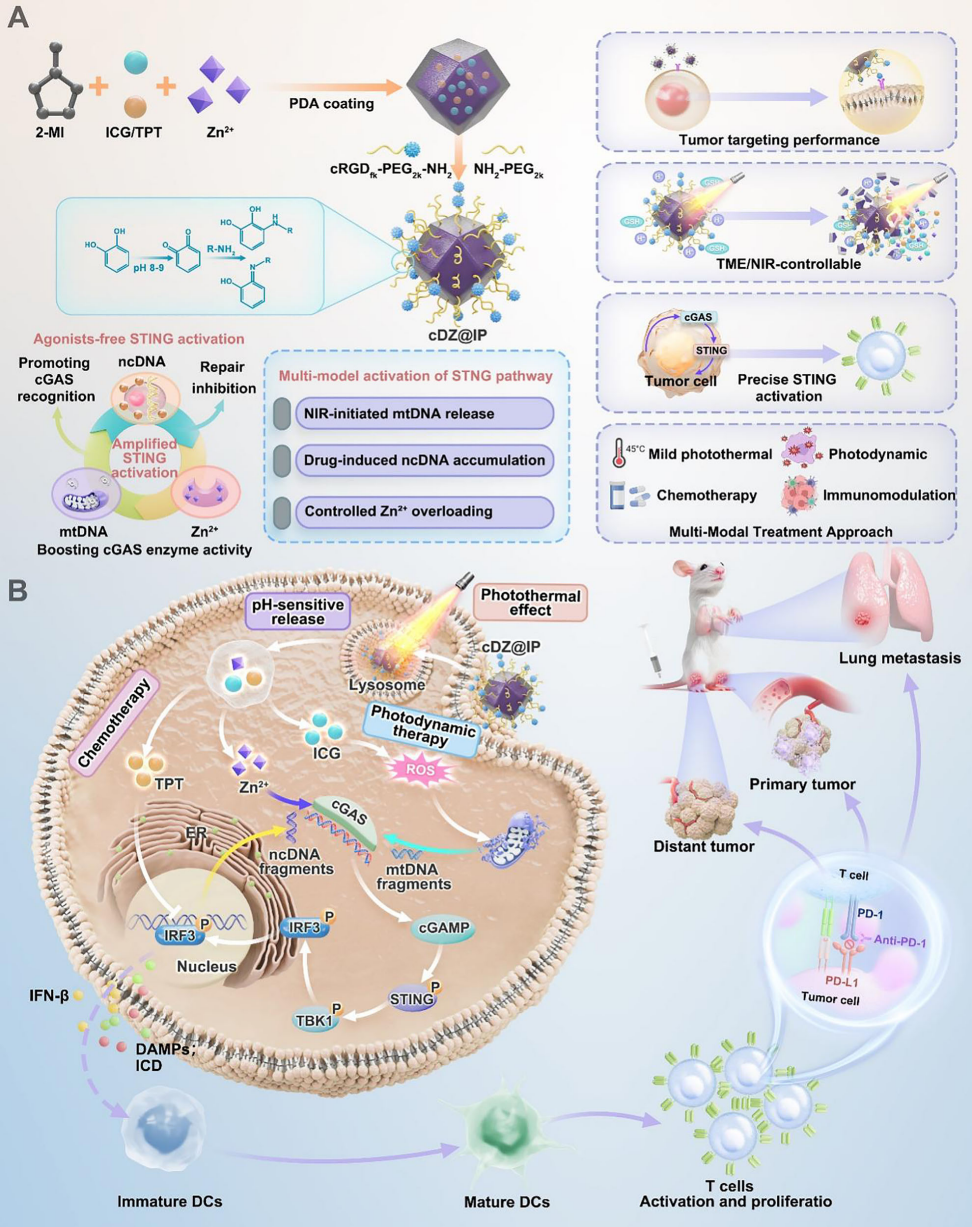
Schematic diagram of the synthesis of cDZ@IP nanoagonist and tumor therapy mechanism. (A) Schematic diagram of the synthetic process of the cDZ@IP nanoagonist; (B) the anti‐tumor mechanism diagram of cDZ@IP‐mediated activation of the cGAS–STING signaling pathway to initiate systemic antitumor immunity and enhance the efficacy of immune checkpoint blockade therapy, thereby suppressing both primary and metastatic tumors.

Herein, a multifunctional endogenous/exogenous dual‐responsive nano‐metabolite–based nanoagonist, cRGD‐PDA/ZIF8@ICG/TPT (cDZ@IP), was developed for multimodal amplification of STING activation and immunomodulation synergistic therapy against tumors (Scheme 1). The nanoagonist consists of a Metal organic framework (MOF) core (ZIF8@ICG/TPT; Z@IP) co‐loaded with TPT and ICG, and further modified with polydopamine (PDA) and cRGD_fk_‐PEG_2k_‐NH_2_ for enhanced tumor targeting. cDZ@IP exhibits excellent tumor specificity and potent STING activation capacity, offering strong potential for effective cancer nano‐immunotherapy. Once internalized, intracellular H^+^/GSH and NIR laser irradiation serve as endogenous and exogenous triggers, respectively, initiating nanoagonist disassembly at the tumor site. Subsequently, ICG generates high ROS levels under NIR light, causing mitochondrial oxidative damage and the release of mtDNA. Concurrently, TPT enhances cytosolic DNA accumulation by inhibiting nuclear DNA repair. Elevated intracellular Zn^2^^+^ further enhances cGAS enzymatic activity. These combined effects synergistically activate the cGAS–STING pathway, while photothermal effects induce robust ICD. As a result, STING signaling is upregulated, leading to increased cytokine and IFN‐β secretion, dendritic cell maturation, and CD8^+^ T cell infiltration. In vivo studies using distant tumor and lung metastasis models demonstrated that under NIR irradiation, cDZ@IP significantly inhibited both primary and metastatic tumor growth and delayed lung metastasis. Collectively, this work presents a nano‐metabolomics–enabled immune activation strategy that addresses the limitations of traditional STING agonists by integrating precise STING activation, mPTT, and immunomodulation. In this strategy, nano‐metabolomics involves the synergistic roles of three key components: the photosensitizer ICG, the chemotherapy drug TPT, and Zn^2+^. This approach provides a conceptual framework for the rational design of therapeutic metabolites and establishes a new paradigm at the intersection of nanomedicine and cancer immunotherapy.

## 2 Results and Discussion

### 2.1 Activation and Upregulation of the cGAS‐STING Pathway in Colorectal Cancer

**FIGURE 1:**
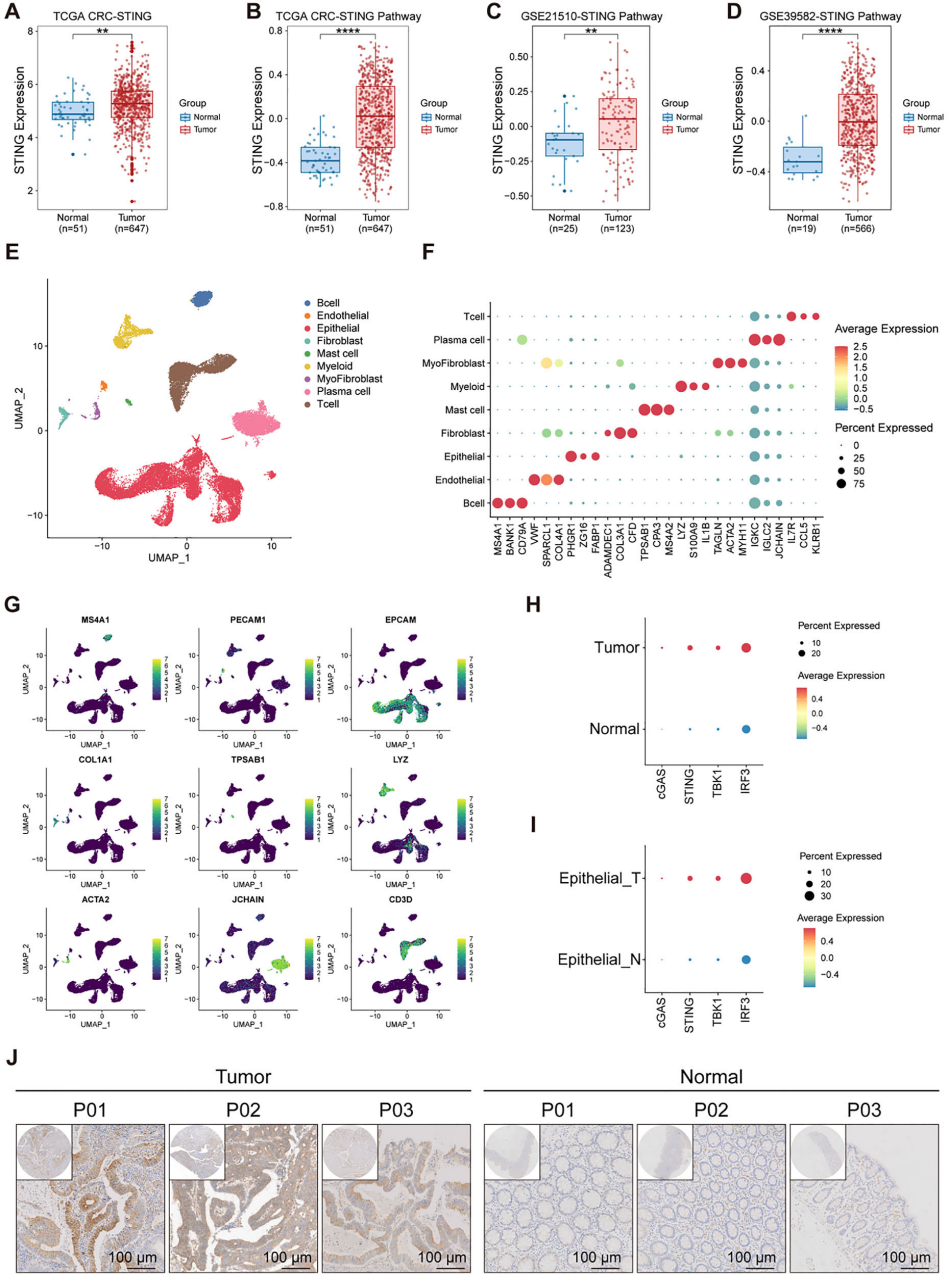
Differential Expression of STING in Cancerous and Normal Epithelium. (A) The expression profile of STING in the TCGA cohort; (B‐D) Assessment of STING pathway activity scores in the TCGA (B), GSE21510 (C), and GSE39582 (D) datasets; (E) A UMAP visualization that identified 9 major cell types exhibited in CRC and normal tissues; (F) Dot plot exhibiting the expression level of key marker genes across the cell types; (G)UMAP visualization of canonical marker gene expression for identification of major cell types; (H) Dotted lines represented P‐adjusted value 0.4 and < −0.4 corresponding to Tumor and Normal cells, respectively; (I) Dotted lines represented P‐adjusted value 0.4 and < −0.4 corresponding to Tumor Epithelial and Normal Epithelial cells, respectively; (J) Immunohistochemical analysis of STING in human CRC and normal tissues.

We investigated the expression levels of the cGAS–STING signaling pathway in colorectal cancer (CRC), including colon adenocarcinoma and rectal adenocarcinoma (COADREAD). The expression of STING pathway components in CRC tissues was analyzed using data from The Cancer Genome Atlas (TCGA), as well as the GSE21510 and GSE39582 datasets. The results revealed that STING mRNA expression was significantly higher in CRC tissues compared to normal colorectal tissues (Figure 1A). In addition, the mRNA levels of cGAS, TBK1, and IRF3 were also elevated in tumor tissues (Figure S1). Pathway activity scoring further demonstrated that the overall activity of the STING signaling pathway was markedly higher in CRC than in normal tissues (Figure 1B‐D). Next, we performed a paired analysis of five CRC tumor samples and their matched adjacent normal tissues using a publicly available single‐cell RNA sequencing dataset GSE166555. Based on the differential expression of established lineage markers (Figure S2), a Uniform Manifold Approximation and Projection (UMAP) visualization was used to identify and categorize 9 major cell types in CRC and adjacent normal tissues, as shown in Figure 1E‐G. Notably, cGAS, STING, TBK1, and IRF3 were predominantly enriched in epithelial cells (Figure S3). Compared to normal tissues, the expression of these STING‐related genes was significantly upregulated in tumor tissues (Figure 1H). Moreover, epithelial cells in CRC exhibited significantly higher expression levels of STING‐related genes than those in normal epithelial cells (Figure 1I). Finally, we collected human CRC tissue samples and conducted histological analysis. Immunohistochemical staining revealed elevated STING protein expression in CRC tissues (Figure 1J), suggesting that targeting the cGAS–STING pathway may represent a potential therapeutic strategy for CRC.

### 2.2 Preparation and Characterization of cDZ@IP NPs

**FIGURE 2:**
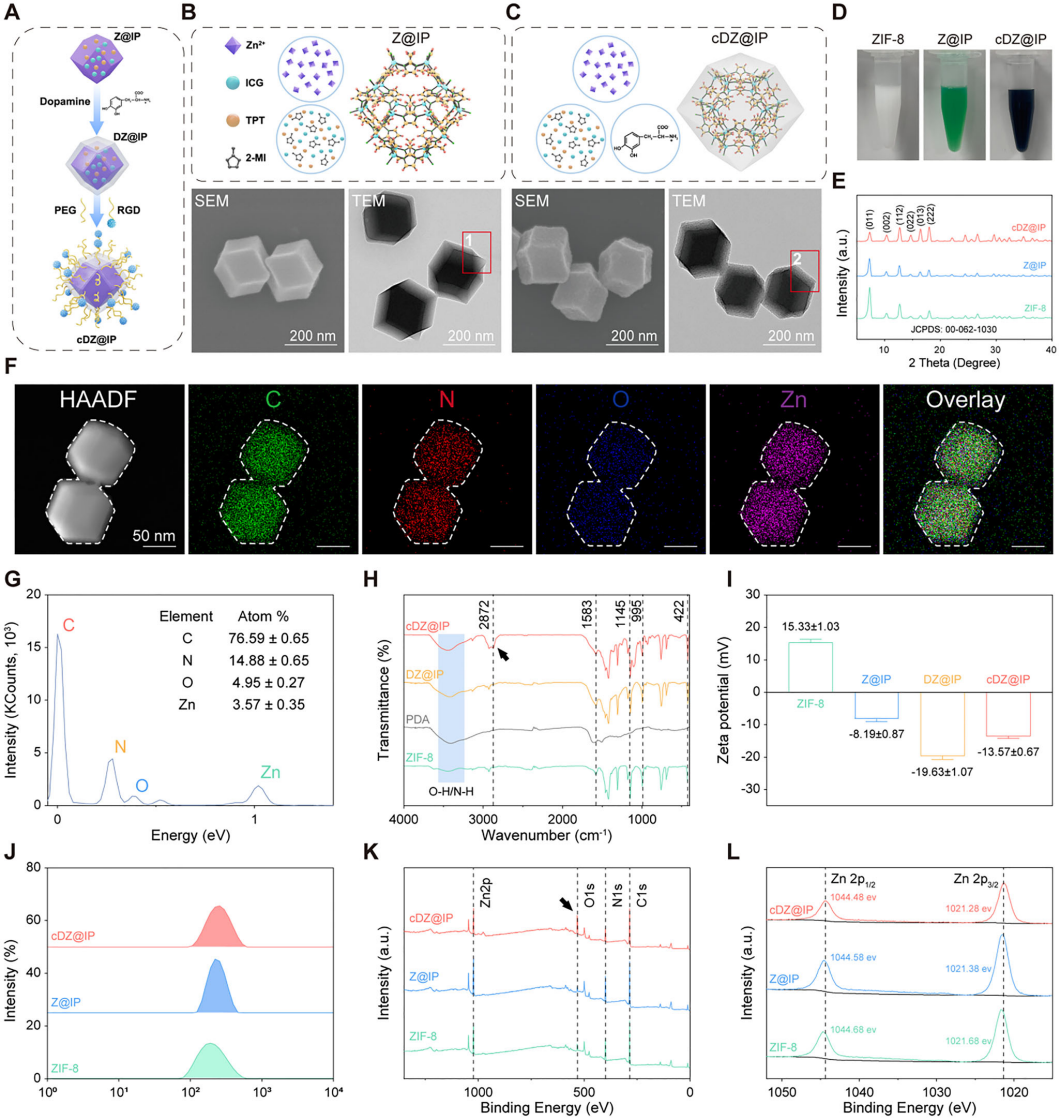
Structural characterizations of cDZ@IP nanoagonists. (A) Schematic diagram of the preparation of the cDZ@IP NPs; (B, C) Schematic models and corresponding SEM and TEM photographs of Z@IP (B) and cDZ@IP (C) NPs. (Insets depict molecular and structural representations of the corresponding materials, highlighting the steric‐hindrance effect induced by dopamine coating); (D) Solubility of ZIF‐8, Z@IP, and cDZ@IP NPs in water; (E) XRD patterns of ZIF‐8, Z@IP, and cDZ@IP NPs; (F) HAADF‐STEM and elemental mapping images of C, N, O, and Zn in the cDZ@IP NPs; (G) EDS spectrum and elemental composition of cDZ@IP NPs; (H) FTIR spectra of ZIF‐8, PDA, DZ@IP, and cDZ@IP; (I) Zeta potential of ZIF‐8, Z@IP, DZ@IP, and cDZ@IP (n = 3); (J) Hydrodynamic size distribution of ZIF‐8, Z@IP, and cDZ@IP measured by DLS (PDI polydisperse index); (K, L) XPS spectrum (K) and Zn 2p (L) of ZIF‐8, Z@IP, and cDZ@IP. Data are presented as mean ± standard deviations from a representative experiment (n = 3 independent samples). *P* values were analyzed by one‐way ANOVA with Tukey's multiple comparisons post hoc test.

Zeolitic imidazolate framework‐8 (ZIF‐8) and Z@IP NPs were synthesized via a one‐step solvothermal method [52]. The surface of Z@IP was sequentially functionalized with PDA to form DZ@IP, followed by cyclo(RGD‐DPhe‐K)‐(Polyethylene glycol)‐2000‐amino (cRGD_fk_‐PEG_2k_‐NH_2_) conjugation, yielding the zinc‐based nanoagonist (cDZ@IP) (Figure 2A). Specifically, the strong interaction between Zn^2^^+^ and 2‐MI facilitates the formation of regular dodecahedral structures with uniform distribution in ZIF‐8 (Figure S4). Similarly, scanning electron microscopy (SEM) and transmission electron microscopy (TEM) images reveal that Z@IP exhibits the same dodecahedral morphology, indicating that the loading of ICG and TPT did not affect the original structure of the ZIF‐8 (Figure 2B). The distribution of Z@IP is shown in Figure S5. UV–vis absorption spectra shown that Z@IP NPs exhibited a significant redshift of the characteristic absorption peak of ICG from 778 to 850 nm, suggesting aggregation of ICG at high local concentrations within ZIF‐8 NPs (Figures S6 and S7). As shown in Figure S8, ICG loading efficiency and entrapment efficiency in Z@IP is 14.99% ± 0.0002% and 99.97% ± 0.0018%, respectively. Furthermore, the encapsulation efficiencies of TPT within the Z@IP NPs were calculated to be ∼98.93% ± 0.012% (loading amount: ∼4.94 wt.%, Figure S9), as determined by high‐performance liquid chromatography (HPLC). These outcomes demonstrate the successful loading of ICG and TPT into the synthesized ZIF‐8.

After modification with PDA, the surface of DZ@IP became rougher (Figure 2C, Figure S10). Interestingly, the changes in ZIF‐8 during the modification process can be easily distinguished by their color (Figure 2D). The x‐ray diffraction (XRD) patterns suggested that the crystal structure of ZIF‐8 was slightly changed after the formation of PDA shell (Figure 2E). This is attributed to the partial amorphization of ZIF‐8 induced by the coordination interactions between dopamine and Zn^2+^, which is consistent with the observations in TEM (Figure S11) [53]. In addition, high‐angle annular dark‐field scanning T energy‐dispersive x‐ray spectroscopy (HAADF‐STEM‐EDS) mapping images reveal the uniform distribution of Zn, O, N, and C elements across the cDZ@IP (Figure 2F,G). The surface modification of PDA and cRGD on cDZ@IP was first investigated by Fourier‐transform infrared spectroscopy (FTIR) (Figure 2H). Compared with DZ@IP, cDZ@IP exhibited additional absorption features, including the peaks at 1583 cm^−1^ (stretching vibration of C═N) and between 1145 and 995 cm^−1^ (absorptions of C─N) was originated from ZIF‐8 [54]. The absorption band between 3100 and 3600 cm^−1^ corresponded to the N─H and O─H stretching vibration in the PDA; and the peak at 2872 cm^−1^ was characteristic absorption peak of cRGD. However, due to the limited specificity of FTIR signals, further characterization was performed. To avoid interference from ICG and TPT, UV–vis spectroscopy was conducted using cDZ and DZ samples (Figure S12). An additional absorption feature was observed in cDZ compared to DZ, providing more direct evidence for the successful conjugation of cRGD. In addition, the zeta potential changed significantly after surface modification (Figure 2I), further indicating successful functionalization of the nanoparticles. The average sizes of ZIF‐8, Z@IP, and cDZ@IP NPs were determined to be 186.1 ± 2.7 nm, 227.3 ± 3.4 nm, and 248.3 ± 5.9 nm, respectively (Figure 2J). In summary, these results support the successful coating of PDA and conjugation of cRGD onto the nanoparticle surface.

The x‐ray photoelectron spectroscopy (XPS) confirmed the existence of Zn, O, N, and C elements (Figure 2K), which consistent with the TEM result. The individual analysis of zinc element showed that the binding energy of Zn 2p was decreased in cDZ@IP NPs compared with ZIF‐8 (Figure 2L), indicating a looser binding of zinc with the components in cDZ@IP NPs, which facilitates the release of Zn during the treatment process.

### 2.3 Stimuli‐Responsive Performance of cDZ@IP

**FIGURE 3:**
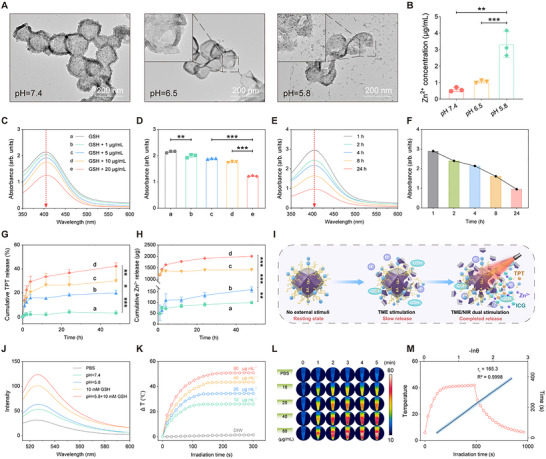
Characterization of the material properties and performance of cDZ@IP NPs. (A) TEM images of cDZ@IP NPs at pH 7.4, 6.5, and 5.8; (B) The concentration of Zn^2+^ in the supernatant after incubation of cDZ@IP NPs for 24 h under different pH conditions (n = 3); (C) Concentration‐dependent GSH (3 mm) depletion by cDZ@IP NPs; (D) Statistical analysis of the depletion of GSH after incubation with cDZ@IP NPs at different concentrations (n = 3); (E, F) Time‐dependent GSH (3 mm) depletion by cDZ@IP NPs. (G, H) TPT (G) and Zn^2+^ (H) cumulative release curves of cDZ@IP NPs under different conditions (a: control, b: pH 5.8, c: pH 5.8 + 10 mm GSH, and d: pH 5.8 + 10 mm GSH + L, n = 3); (I) Schematic illustration of the pH‐responsive release of TPT, ICG, and Zn^2^^+^ from cDZ@IP NPs under laser irradiation; (J) The singlet oxygen (^1^O_2_) yield of cDZ@IP NPs + laser in PBS under different conditions; (K) Concentration‐dependent photothermal performance of cDZ@IP NPs under laser irradiation; (L) Thermographs of different cDZ@IP concentrations over time; (M) Curves showing temperature variation in cDZ@IP nanoparticles, both with and without 808 nm NIR exposure at 1.0 W cm^−2^; Fitting linearly the −lnθ versus time for cDZ@IP NPs. Data are presented as mean ± standard deviations from a representative experiment (n = 3 independent samples). *P* values were analyzed by one‐way ANOVA with Tukey's multiple comparisons post hoc test. ***p < 0.001; **p < 0.01; *p < 0.05.

The stimuli‐responsive degradation of the cDZ@IP NPs was evaluated under endogenous tumor microenvironment (TME). Under acidic conditions simulating the TME (pH 7.4, 6.5, 5.8), TEM revealed progressive structural changes: surface etching occurred at pH 7.4 and 6.5, while significant degradation occurred at pH 5.8 (Figure 3A). Consistent with this, ion release at pH 5.8 was ∼5.69‐fold higher than at pH 7.4 (Figure 3B), confirming TME‐triggered disintegration. cDZ@IP NPs also depleted glutathione (GSH), a key TME antioxidant, via PDA‐mediated thiol reactions [55]. Specifically, we assessed GSH depletion by cDZ@IP NPs using 5,5'‐dithiobis‐(2‐nitrobenzoic acid) (DTNB), a reagent that forms a yellow product (λmax = 412 nm) upon reaction with GSH. cDZ@IP NPs exhibited concentration‐dependent GSH depletion (Figure 3C). Treatment with 20 µg mL^−^^1^ cDZ@IP NPs for 2 h reduced GSH content by ∼43% (Figure 3D). Time‐dependent depletion was also observed (Figure 3E,F). Therefore, the depletion of GSH by cDZ@IP prevents ROS scavenging, resulting in enhanced anti‐tumor efficacy.

Furthermore, we investigated the light‐responsive drug (ICG, TPT) release and Zn^2^^+^ extraction from cDZ@IP under a simulated tumor TME conditions (mild acidity, high GSH). Under NIR irradiation, cumulative TPT release reached 42% ± 3.01% within 48 h, significantly exceeding levels in the dark (3% ± 1.97%) (Figure 3G). Concurrently, the combined endogenous (acidic pH, high GSH) and exogenous (NIR) stimuli accelerated Zn^2^^+^ release, achieving 2007.5 ± 22.5 µg by 48 h (Figure 3H). These results demonstrate that TME/NIR‐triggered decomposition of cDZ@IP enables responsive unloading of photosensitizers, chemotherapeutics, and metal ions (Figure 3I). Crucially, while ICG's photodynamic activity is potentially quenched within intact cDZ@IP, its dissociation under triple stimulation (acidic pH + GSH + NIR) markedly enhanced singlet oxygen (^1^O_2_) generation, as quantified by SOSG assay (Figure 3J, Figure S13). This is attributed to improved contact efficiency between liberated ICG and oxygen upon degradation of the PDA shell and ZIF‐8 core. ROS production further correlated positively with ICG concentration, irradiation duration, and NIR power intensity (Figure S14). This tumor‐specific, NIR‐enhanced release mechanism facilitates in situ activation of antitumor immunity.

The photothermal conversion performance of cDZ@IP was evaluated under 808 nm NIR irradiation. Both cDZ@P and cDZ@IP exhibited concentration‐dependent temperature increases, with ICG‐incorporated cDZ@IP showing significantly enhanced efficiency (Figure 3K,L; Figure S15). Under identical irradiation (1.0 W cm^−2^), ICG‐incorporated cDZ@IP demonstrated significantly enhanced photothermal conversion compared to PBS controls (ΔT ≈ 1.4°C). A clear laser‐power‐dependent temperature response was also observed (Figure S16). Crucially, cDZ@IP maintained consistent heating efficacy through four on/off NIR cycles, confirming excellent photothermal stability (Figure S17). The calculated photothermal conversion efficiency (η = 51.58%, Figure 3M) further validates its high performance.

### 2.4 Cellular Uptake and Antitumor Effect of cDZ@IP NPs

**FIGURE 4:**
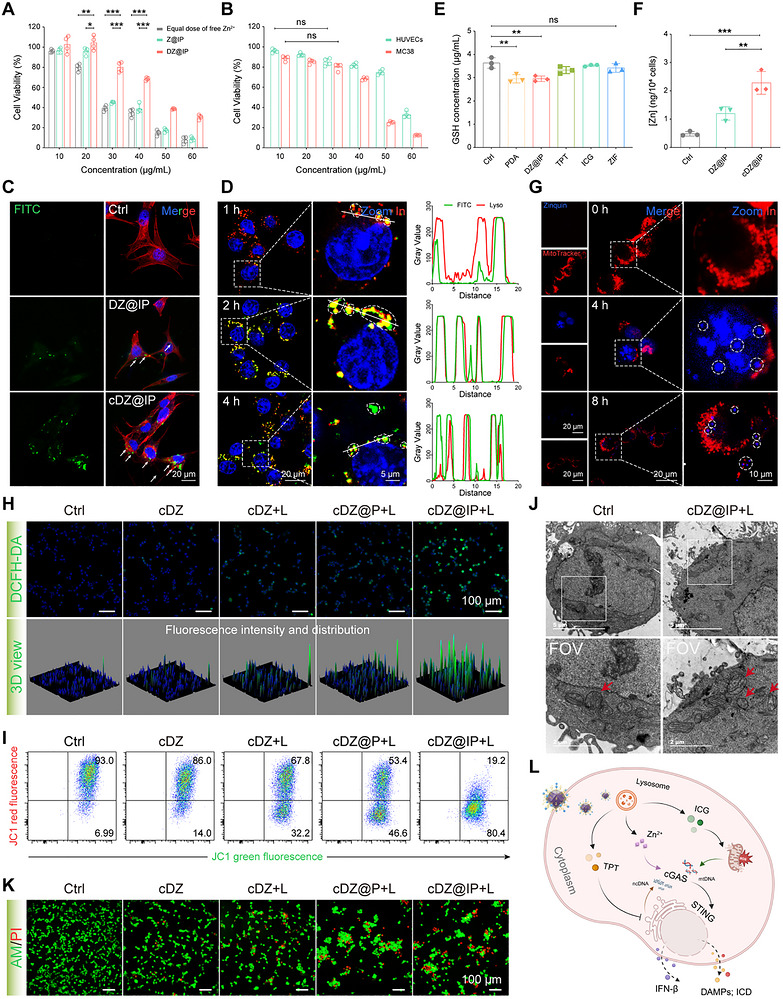
Antitumor effect of cDZ@IP nanoagonists under laser irradiations. (A) Cell viability of MC38 cells after 24 h coincubation with free Zn^2+^ (equal Zn dose), Z@IP and DZ@IP NPs (n = 4); (B) Cell viability of HUVECs and MC38 cells after 24 h coincubation with DZ@IP NPs (n = 4); (C) Endocytosis effect after co‐incubation of FITC‐labeled DZ@IP or cDZ@IP NPs with MC38 cells (Green channel: FITC‐labeled DZ@IP or cDZ@IP NPs, Red channel: Cytoskeleton, Blue channel: Hoechst33342, scale bar: 20 µm); (D) Time‐dependent lysosomal escape behavior of cDZ@IP (Green channel: FITC‐labeled cDZ@IP NPs, Red channel: lysosomes, Blue channel: Hoechst33342, scale bars: 20 µm; inset shows enlarged views, scale bars: 5 µm). The right panels present the corresponding line‐scan fluorescence intensity profiles of FITC and lysosomal signals; (E) Intracellular GSH levels in MC38 cells after treatment with different formulations (n = 3); (F) Quantification of Zn^2^^+^ levels in MC38 cells after different treatments (n = 3); (G) Mitochondrial targeting of Zinc ion Probe after co‐incubation of cDZ@IP NPs with MC38 cells for different times (Red channel: mitochondrion, Blue channel: Zinquin, scale bar: 20 µm; inset shows enlarged views, scale bars: 10 µm); (H) Intracellular ROS levels of MC38 cells were detected by DCFH‐DA probe (green) after different treatments (material concentration: 20 µg mL^−1^, scale bar: 100 µm, inset shows 3D fluorescence); (I) Flow cytometry analysis of mitochondrial membrane potential of MC38 cells after various treatments with a material concentration of 20 µg mL^−1^; (J) Bio‐TEM images of mitochondrial morphological changes in MC38 cells treated without and with cDZ@IP + L (Scale bars: 5 µm (up) and 2 µm (down)); (K) Live/death staining of MC38 cells after different treatments (Green channel: Calcein‐AM, Red channel: PI, material concentration: 20 µg mL^−1^, scale bar: 100 µm); (L) Schematic illustration of the intracellular mechanism of cDZ@IP nanoagonists. Created with BioRender.com. Data are presented as mean ± standard deviation from a representative experiment (n ≥ 3 biologically independent samples). *P* values were analyzed by one‐way ANOVA with Tukey's multiple comparisons post hoc test. ***p < 0.001; **p < 0.01; *p < 0.05; ns, no significant.

Before conducting cell experiments, we first evaluated the cytotoxicity of nanoagonists. As shown in Figure 4A, free Zn^2+^ exhibited relatively higher cytotoxicity toward MC38 cells compared with the nanoparticle formulations. Z@IP nanoparticles showed moderate cytotoxicity, whereas PDA‐coated DZ@IP displayed significantly improved cytocompatibility, suggesting that Zn^2+^ may contribute to the observed cytotoxicity and that PDA modification can partially alleviate Zn^2^^+^‐related nonspecific toxicity. Furthermore, we investigated the effects of DZ@IP nanoparticles on different cell lines. The results showed that DZ@IP NPs caused negligible toxicity toward both human umbilical vein endothelial cells (HUVECs) and MC38 cells at the working concentration of 20 µg mL^−1^ (Figure 4B). Subsequently, confocal laser scanning microscopy (CLSM) and flow cytometry were employed to evaluate the cellular uptake efficiency of cDZ@IP NPs in MC38 tumor cells. As expected, MC38 tumor cells treated with cDZ@IP for 6 h exhibited a higher intensity of green fluorescence compared to those in the DZ@IP treatment group (Figure 4C). This enhancement can be attributed to the tumor‐targeting ligand cRGD_fk_‐PEG_2k_‐NH_2_, which was introduced onto the surface of DZ@IP nanoparticles to promote cellular internalization. To further verify this observation, quantitative analysis was performed using flow cytometry. As shown in Figure S18, the mean fluorescence intensity (MFI) of the cDZ@IP‐treated cells was approximately twofold higher than that of the DZ@IP group, indicating that cRGD modification significantly improves the cellular uptake efficiency of the nanoparticles. Following cellular internalization, nanoparticles are typically trafficked into lysosomes, where they may undergo degradation or exocytosis. As demonstrated in Figure 3A,B, the cDZ@IP NPs exhibit pronounced pH‐responsive degradation behavior, with accelerated structural collapse and Zn^2+^ release under acidic conditions that mimic the lysosomal environment (pH ≈ 5.5–6.0). Such acid‐responsive disintegration is associated with the protonation of imidazole groups in the ZIF‐8 framework, which may facilitate lysosomal escape [56]. To further investigate the intracellular trafficking behavior of the nanoagonists, lysosomal colocalization analysis was performed. As shown in Figure 4D, the green fluorescence of cDZ@IP nanoparticles initially shows strong overlap with the red fluorescence of lysosomes after 2 h of incubation, indicating that the nanoparticles are effectively internalized and accumulate within lysosomes following endocytosis. Notably, the degree of fluorescence overlap decreases markedly at 4 h, accompanied by a more dispersed distribution of nanoparticle fluorescence in the cytoplasm. This observation suggests that a fraction of the nanoparticles gradually escape from lysosomes after internalization. These results, together with the acid‐responsive degradation behavior of ZIF‐8 under lysosome‐mimicking acidic conditions, support the lysosomal escape capability of cDZ@IP nanoparticles. This observation further confirms that cDZ@IP nanoagonists can successfully escape from lysosomes and exert therapeutic effects within the cytoplasm of cancer cells, thereby laying the foundation for subsequent selective degradation and NIR‐induced STING activation.

Intracellular GSH depletion and Zn^2+^ overload are critical for immune activation therapy. Therefore, the GSH levels in MC38 cells treated with various components were evaluated using a reduced glutathione assay kit. Compared to the control group, no significant changes in GSH levels were observed in cells treated with TPT, ICG, or ZIF‐8. In contrast, treatment with PDA and DZ@IP NPs significantly reduced intracellular GSH levels (Figure 4E). These findings suggest that DZ@IP effectively depletes the antioxidant GSH in tumor cells. In addition to investigating the effect of the degradable nanoagonist on GSH levels, its ability to induce intracellular Zn^2^^+^ overload was also examined. First, the concentration of Zn^2^^+^ in MC38 cells after treatment was measured using inductively coupled plasma mass spectrometry (ICP‐MS). Notably, the Zn^2^^+^ level in MC38 cells incubated with cDZ@IP was significantly elevated (Figure 4F), which can be attributed to the targeting moiety of the nanoparticles. Furthermore, the Zn^2^^+^ indicator Zinquin was employed to visualize the localization of intracellular free Zn^2^^+^ (Figure 4G). At 4 h, blue fluorescence (representing free Zn^2^^+^) was observed to accumulate in the cytoplasm of MC38 cells, and by 8 h, it gradually localized to the mitochondria, indicating the specific accumulation of Zn^2^^+^ within cancer cells.

To explore the therapeutic mechanism of cDZ@IP NPs, the intracellular ROS level of MC38 tumor cells was assessed. MC38 cells were treated with different formulations at a concentration of 20 µg mL^−1^. Notably, only a weak green fluorescence signal of ROS was observed in the cDZ treatment group. Upon NIR irradiation, the ROS signal intensity increased in both the cDZ + L and cDZ@P + L groups; however, the difference between these two groups was not significant. This phenomenon may be attributed to Zn^2^^+^ overload due to scaffold degradation under NIR irradiation, along with the therapeutic effect of TPT. Furthermore, the strongest ROS signal was detected in the cDZ@IP + L group, with a 38% increase in ROS generation efficiency compared to the cDZ@P + L group. This enhancement is likely due to the potent intracellular ROS production induced by ICG (Figure 4H). Furthermore, a similar trend of ROS signal enhancement was observed by flow cytometry (Figure S19). Compared to the control group, the cDZ treatment group also induced ROS signal generation, which may be attributed to the surface‐coated PDA depleting intracellular GSH levels and subsequently reducing the antioxidant capacity of tumor cells.

A series of studies have indicated that excessive intracellular ROS can lead to mitochondrial damage and cell apoptosis [57]. Given that mitochondrial membrane potential (MMP) is typically sensitive to ROS accumulation within mitochondria, JC‐1 dye was used as a probe to assess MMP in MC38 cells after five different treatments. MC38 cells were treated with different formulations at a concentration of 20 µg mL^−1^. When MMP is high, JC‐1 aggregates and emits red fluorescence, whereas under low MMP conditions, it remains as monomers and emits green fluorescence. The shift from red to green fluorescence can be considered an indicator of mitochondrial damage. As shown in Figure 4I, the cDZ@IP + L treatment group exhibited the strongest green fluorescence signal and the weakest red fluorescence signal compared to the other groups, indicating the lowest mitochondrial membrane potential and the most severe mitochondrial damage (Figure S20). Fluorescence imaging further confirmed these findings (Figure S21). Furthermore, Bio‐TEM observations revealed swollen mitochondria and enlarged cavities in the treated cells, which were distinct from those observed in the control samples (Figure 4J).

Next, a series of tests were conducted to evaluate the effects of different treatments on cell viability. MC38 cells were treated with different formulations at a concentration of 20 µg mL^−1^. CCK‐8 assay results showed that, compared to cDZ and cDZ@P, MC38 cells incubated with cDZ@IP and exposed to NIR irradiation exhibited a sharp decline in cell viability (Figure S22). This effect was likely due to the combined impact of oxidative stress‐induced damage and TPT release. To visually assess the antitumor efficacy of these treatments, MC38 cells were stained with Calcein‐AM and propidium iodide (PI) to distinguish live cells from dead cells. The captured images revealed a significant increase in red fluorescence (dead cells) in the cDZ@IP + L group, whereas the control, cDZ, and cDZ + L groups mainly consisted of viable green fluorescent cells (Figure 4K). To further characterize the cellular response to different treatments, Annexin V‐FITC/PI staining followed by flow cytometry was performed to analyze the cell death profiles. As shown in Figure S23, treatment with cDZ@IP + L significantly increased the proportion of Annexin V‐positive cells compared with the other groups. Specifically, both early apoptotic (Q3) and late apoptotic (Q2) populations were markedly elevated, with the total percentage of apoptotic/late apoptotic cells increasing from 5.36% in the control group to 77.3% after cDZ@IP + L treatment. These results indicate that cDZ@IP‐mediated therapy effectively induces pronounced cancer cell death in vitro. It should be noted that apoptosis represents one of the observable forms of treatment‐induced cell death under these experimental conditions rather than the sole mechanism of tumor cell elimination. Overall, the results demonstrate that cDZ@IP exhibits markedly enhanced in vitro antitumor efficacy through multimodal amplification mechanisms (Figure 4L).

### 2.5 In Vitro cGAS‐STING Activation and ICD Effects Induced by cDZ@IP NPs Under NIR Laser Exposure

**FIGURE 5:**
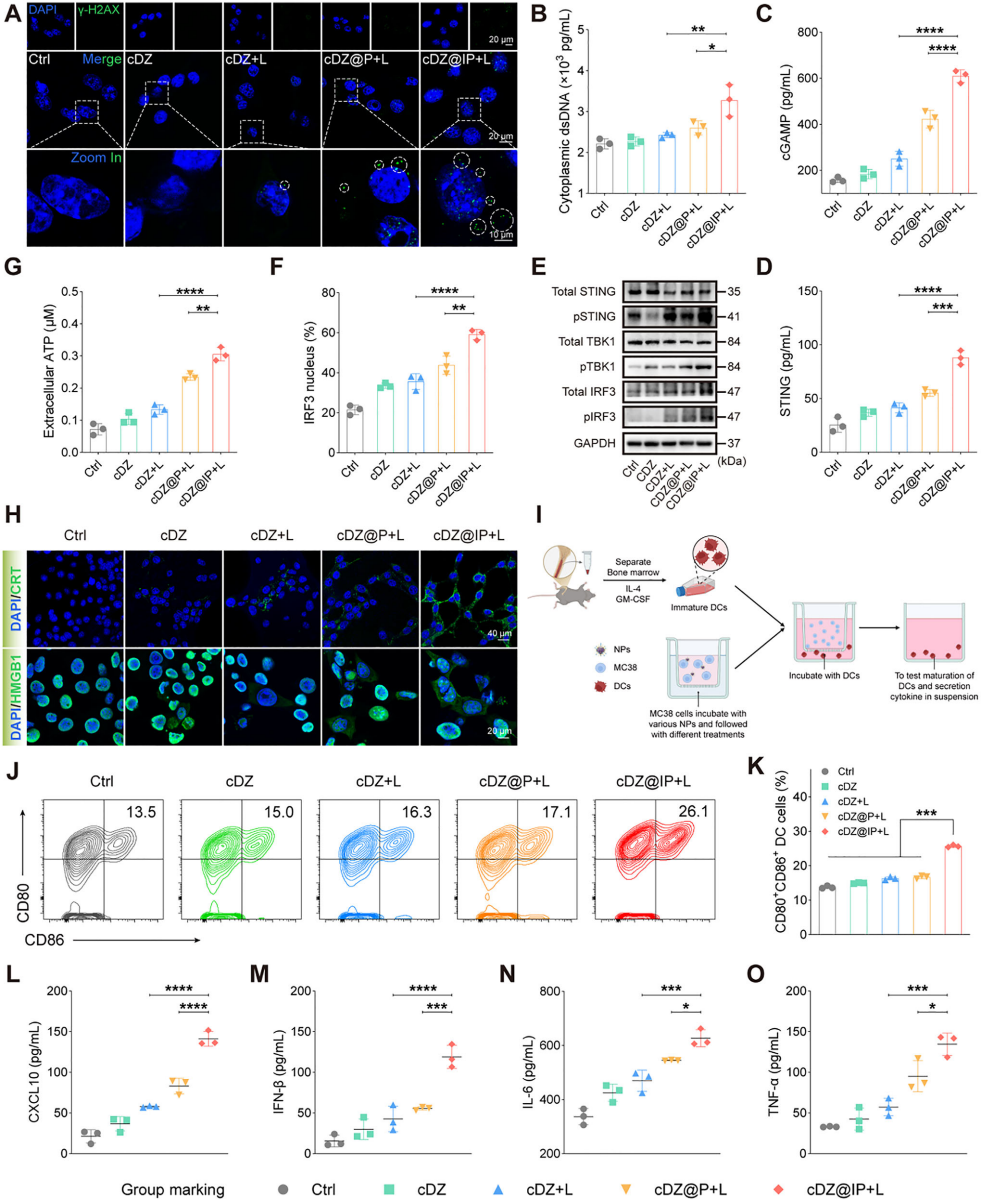
The antitumor mechanism of cDZ@IP nanoagonists under laser irradiations. (A, B) DNA damage (A) and quantitative analysis (B) of intracellular fluorescence intensity in MC38 cells co‐incubated with different treatments (Green channel: γ‐H2AX, Blue channel: Hoechst33342, material concentration: 20 µg mL^−1^, scale bar: 20 µm; inset shows enlarged views, scale bars: 10 µm; n = 3); (C) The concentrations of cGAMP in MC38 cells treated with different nanoagonists (n = 3); (D) The concentrations of STING in MC38 cells treated with different nanoagonists (n = 3); (E) Western blot of the expression of STING, TBK1, IRF3, and their phosphorylated forms in various groups; (F) Quantitative analysis of IRF3 nuclear translocation in MC38 cells under different treatments, showing the percentage of IRF3 localized in the nucleus (n = 3); (G) Detection of ATP of MC38 cells treated with different nanoagonists (n = 3); (H) Fluorescence microscopy images of subcellular localization of CRT exposure and HMGB1 distribution of MC38 cells after different treatments (Scale bar: 40 and 20 µm); (I) The diagram of the transwell system experiment. Created with BioRender.com; (J, K) Flow cytometry analysis (J) of matured DCs (CD11c^+^CD80^+^CD86^+^) and corresponding quantitative analysis (K) after different treatments (n = 3); (L‐O) Secretion levels of inflammatory factors CXCL10 (L), IFN‐β (M), IL‐6 (N), and TNF‐α (O) in MC38 cells after different treatments were measured by ELISA kits (n = 3). Data are presented as mean ± standard deviations from a representative experiment (n = 3 biologically independent samples). *P* values were analyzed by one‐way ANOVA with Tukey's multiple comparisons post hoc test. ****p < 0.0001, ***p < 0.001; **p < 0.01; *p < 0.05.

Recent studies have shown that recognizing damaged mtDNA in the cytoplasm serves as an endogenous stimulus for activating the cGAS‐STING pathway [58]. Here, as previously described, ROS generated by cDZ@IP in mitochondria can oxidize mtDNA and promote its leakage into the cytoplasm, enabling agonist‐free activation of cGAS‐STING under NIR irradiation. Additionally, recent reports suggest that chemotherapeutic agents such as camptothecin (CPT) and etoposide can lead to the release of fragmented DNA into the cytosol, thereby stimulating intrinsic STING‐dependent activity [31, 59]. To investigate this, the expression of the DNA damage biomarker γ‐H2AX in MC38 cells was observed using CLSM. MC38 cells were treated with different formulations at a concentration of 20 µg mL^−1^. Cells treated with cDZ@IP + L exhibited significantly stronger green fluorescence intensity compared to those treated with other methods, both in the nucleus and cytoplasm (Figure 5A). Furthermore, the expression of damaged DNA in tumor cells was quantitatively assessed using the PicoGreen double‐stranded DNA (dsDNA) assay. The results showed that the nanoagonist, upon NIR irradiation, facilitated the rapid cytoplasmic release of damaged DNA (including ncDNA and mtDNA), with levels at least 20.4% higher than those in other groups (Figure 5B). To further distinguish the respective contributions of the chemotherapeutic agent TPT and the nanocarrier system, additional comparative experiments were performed using free TPT at an equivalent dose along with carrier controls (Figure S24). Free TPT treatment induced moderate cytotoxicity and DNA damage, as evidenced by increased γ‐H2AX expression. In contrast, nanoparticle‐delivered TPT (cDZ@P + L) resulted in significantly enhanced cytotoxic effects and DNA damage compared to free TPT at the same dose, indicating improved intracellular delivery efficiency. These results suggest that the nanocarrier system effectively amplifies TPT‐induced DNA damage, thereby contributing to enhanced activation of the cGAS–STING pathway.

Zn^2+^ has been reported to promote the phase separation and enzymatic activity of cGAS, further enhancing immune responses [60]. Therefore, ELISA kits were used to measure the levels of cGAMP and STING in MC38 cells subjected to various treatments. MC38 cells were treated with different formulations at a concentration of 20 µg mL^−1^. The results showed that cDZ@IP + L significantly induced the high expression of cGAMP and STING (Figure 5C,D). This effect may be attributed to oxidative stress‐triggered mtDNA release, ncDNA exposure caused by the chemotherapeutic agent TPT, and the metal‐immune effect of overloaded Zn^2+^. Notably, Western blot analysis revealed that, compared to other groups, the expression of downstream proteins associated with the cGAS‐STING pathway, such as phos‐TBK1 and phos‐IRF3, was significantly upregulated after cDZ@IP + L treatment (Figure 5E, Figure S25). Furthermore, immunofluorescence analysis demonstrated a pronounced increase in nuclear translocation of IRF3 under the same treatment conditions (Figure S26), which was further supported by quantitative analysis (Figure 5F), confirming the functional activation of IRF3 downstream of the cGAS‐STING pathway. Previous studies have also confirmed that tumor cells overexpressing STING exhibit significantly enhanced secretion of CXCL10 and IFN‐β, which play a crucial role in promoting the infiltration of cytotoxic T lymphocytes (CTLs) [61]. To further investigate the levels of inflammatory cytokine secretion under different treatments, the expression of CXCL10 and IFN‐β in MC38 cells was examined. Notably, the levels of CXCL10 and IFN‐β in the cDZ@IP + L group significantly increased to 7‐fold and 7.2‐fold, respectively, compared to the control group (Figure 5L,M). In summary, mtDNA generated from mitochondrial oxidative stress and ncDNA released by the chemotherapeutic agent TPT activated cGAS, subsequently catalyzing the synthesis of the second messenger cGAMP. Simultaneously, Zn^2^^+^ further enhanced cGAS sensitivity to dsDNA, increased enzymatic activity, and strengthened the binding affinity of cGAMP to STING, thereby amplifying STING pathway activation.

There is evidence that oxidative stress, chemotherapeutic agents, and photothermal effects can induce ICD in tumor cells, leading to the release of DAMPs and promoting the transfer of tumor‐associated antigens to dendritic cells (DCs) [62]. This evidence inspired our hypothesis that the nanoparticle‐encapsulated TPT combination could generate a stronger antitumor immune response. To test this hypothesis, we first examined the expression levels of calreticulin (CRT) and high mobility group protein 1 (HMGB1) in treated tumor cells, as these are indispensable biomarkers of tumor cell ICD. MC38 cells were treated with different formulations at a concentration of 20 µg mL^−1^. Immunofluorescence staining data clearly demonstrated strong CRT fluorescence on the plasma membrane of tumor cells treated with DZ@IP + L and cDZ@IP + L. Meanwhile, the release of HMGB1 was confirmed by the reduction of green fluorescent spots in the nuclei of treated cells (Figure 5H). Additionally, under treatment with cDZ@IP NPs, a significant amount of ATP was secreted into the extracellular environment of MC38 cells (Figure 5G). These results indicate that cDZ@IP NPs treatment effectively enhances the immunogenicity of cancer cells. Next, a transwell system was used to co‐incubate BMDCs with MC38 cells for 24 h to further assess the ability of cDZ@IP NPs to induce DC maturation in vitro (Figure 5I). The results showed a significant increase in CD80/CD86‐expressing DCs in the cDZ@IP‐treated group compared to the control group (Figure 5J,K). Furthermore, ELISA analysis was performed to evaluate cytokine secretion by DCs, revealing that IL‐6 and TNF‐α levels in the culture medium were notably elevated in the cDZ@IP + L group (Figure 5N,O). These findings provide strong evidence that the nanoagonist effectively triggers a robust immune response in vitro by enhancing cGAS‐STING activation and ICD effects.

### 2.6 In Vivo Biodistribution and Activation of STING‐Mediated Antitumor Immunotherapy by cDZ@IP

To investigate the in vivo behavior of cDZ@IP NPs, their tumor‐targeting capability, pharmacokinetics, and metabolic pathways were systematically evaluated in MC38 tumor‐bearing mice. The tumor‐targeting capability of cDZ@IP NPs was first assessed using Cy7.5‐labeled nanoparticles following intravenous injection (100 µL, 5 mg kg^−1^). As shown in Figures S27A and S28, cDZ@IP NPs exhibited strong and sustained fluorescence signals at the tumor site, which persisted up to 72 h post‐injection, indicating efficient tumor accumulation and prolonged retention. In contrast, DZ@IP NPs showed weaker tumor localization and more rapid signal attenuation, demonstrating the enhanced tumor‐targeting capability after surface modification. To quantitatively evaluate the pharmacokinetic behavior, the blood circulation profile of cDZ@IP NPs was further analyzed (Figure S29). The results showed that the t_1/2_ of cDZ@IP NPs in the body was 8.66 h. To elucidate the metabolic pathways, ex vivo fluorescence imaging of major organs was first performed (Figure S27B). Notably, strong fluorescence signals were observed in the kidney, indicating that renal clearance plays a major role in nanoparticle elimination. Quantitative analysis of fluorescence intensity in major organs further confirmed this trend (Figure S30A). Consistently, the Zn content detected in urine increased gradually over time (Figure S30B), further verifying that the nanoparticles were predominantly eliminated via the renal excretion pathway. In addition, the kidney‐to‐tumor ratio was significantly reduced in the cDZ@IP group compared with DZ@IP (Figure S30C), indicating decreased renal accumulation and enhanced tumor retention after surface modification. Collectively, these results demonstrate that cDZ@IP NPs possess prolonged blood circulation, enhanced tumor‐targeting capability, and a predominantly renal clearance pathway, providing a favorable pharmacokinetic and metabolic profile for effective in vivo cancer immunotherapy.

**FIGURE 6:**
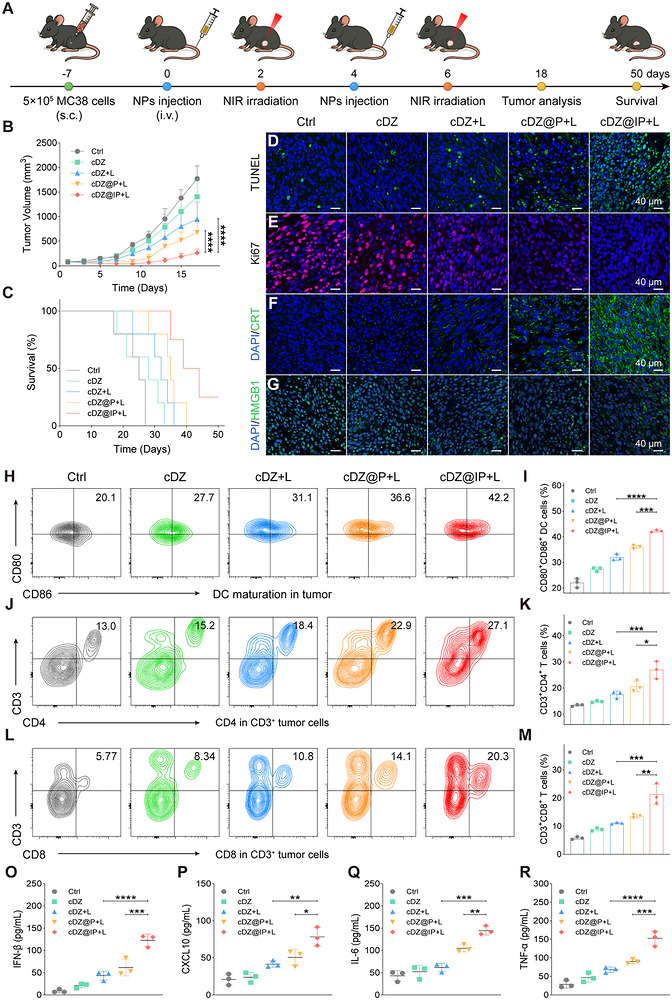
The biodistribution and antitumor immune response of cDZ@IP nanoagonists in MC38 tumor‐bearing mice. (A) Schematic illustration of the treatment schedule in C57BL/6 mice; B Tumor growth curves of mice after different treatments (n = 5); (C) Survival time of tumor‐bearing mice in each group (n = 5); (D‐G) Representative fluorescence images of tumor slices after TUNEL (D), Ki67 (E), CRT (F), and HMGB1 (G) immunofluorescence staining (Green channel: TUNEL, CRT, and HMGB1, Magenta channel: Ki67, Blue channel: DAPI, scale bars: 40 µm); (H‐M) Flow cytometry analysis and quantification of matured DCs (H, I), CD4^+^ T cells (J, K), and CD8^+^ T cells (L, M) in tumors after various treatments (n = 3); (O‐R) The levels of IFN‐β (O), CXCL10 (P), IL‐6 (Q), and TNF‐α (R) in the tumor after different treatments (n = 3). Data are presented as mean ± standard deviation from a representative experiment (n ≥ 3 biologically independent samples). *P* values were analyzed by one‐way ANOVA with Tukey's multiple comparisons post hoc test. ****P < 0.0001, ***P < 0.001; **P < 0.01; *P < 0.05.

Next, the therapeutic efficacy of various nanoparticle agonists in inhibiting tumor growth was evaluated in MC38 tumor‐bearing mice following intravenous administration (100 µL, 5 mg kg^−1^). The tumor treatment protocol is illustrated in Figure 6A. To control the temperature during photothermal therapy, the temperature variation at the tumor site was monitored during NIR irradiation treatment. The results showed that the tumor temperature remained consistently around 45°C (Figure S31), indicating that cDZ@IP NPs consistently exerted a mild photothermal effect. In vivo results demonstrated that the cDZ@IP + L group exhibited the strongest antitumor effect, with an average tumor volume of only 260.6 mm^3^ after 18 days. In contrast, limited tumor suppression was observed in the cDZ@P + L and cDZ + L groups. Meanwhile, the tumor volumes in the PBS control and cDZ groups reached 1771.3 and 1405.9 mm^3^, respectively (Figure 6B). Notably, tumor‐bearing mice treated with cDZ@IP + L exhibited the highest tumor suppression efficacy, with a tumor inhibition rate of approximately 92.15%. Digital photographs of the excised tumors, along with quantitative analysis, revealed that the average tumor weight in the cDZ@IP + L group (∼0.163 g) was only 8.02% of the average tumor weight in the control group (∼2.03 g) (Figures S32 and S33). Moreover, compared to other groups, the cDZ@IP + L treatment significantly prolonged the survival time of tumor‐bearing mice (Figure 6C), further highlighting its superior therapeutic efficacy. Thereafter, H&E staining, TUNEL staining, and Ki67 immunostaining were performed to assess tumor histopathology, apoptosis, and proliferative activity, respectively (Figure S34 and Figure 6D,E). Compared to other treatment groups, the cDZ@IP + L group exhibited the highest level of tumor apoptosis and the lowest proportion of proliferating cells. Meanwhile, no significant changes in body weight were observed in mice during the treatment period (Figure S35), indicating that cDZ@IP exhibits favorable therapeutic efficacy and excellent biocompatibility.

To further elucidate the mechanism underlying the synergistic antitumor effect, we first assessed the expression of key ICD biomarkers—calreticulin (CRT) and high mobility group box 1 (HMGB1)—in tumors from the different treatment groups. Immunofluorescence staining revealed a marked increase in CRT exposure and a significant reduction in HMGB1 signal in the cDZ@P + L and cDZ@IP + L groups (Figure 6F,G), indicating the occurrence of nanoagonist‐induced ICD in vivo. Subsequently, we evaluated dendritic cells (DCs) maturation within the tumor microenvironment by flow cytometry, which showed that 42.2% of the DCs in tumors from the cDZ@IP + L group were mature CD80^+^CD86^+^ DCs—significantly higher than in other groups (Figure 6H,I, and Figure S36). Similar results were observed in the spleen, where the number of mature DCs increased by 2.84‐fold compared to the control group, along with a 2.09‐fold increase in the tumor (Figure S37A,B). Collectively, these findings demonstrate that the combination of the nanoagonist and NIR irradiation effectively induces ICD in tumor cells and promotes DCs maturation both locally and in immune‐related organs, potentially enhancing tumor antigen presentation and the overall antitumor immune response.

Tumor tissues were harvested from euthanized mice to further analyze the mechanism of cDZ@IP‐mediated antitumor therapy through STING activation. Western blot analysis, supported by quantitative densitometric analysis from independent experiments, demonstrated that the phosphorylation levels of STING and IRF3 (p‐STING/STING and p‐IRF3/IRF3) were significantly increased in the cDZ@IP + L group compared to other treatment groups (Figure S38). Furthermore, ELISA kits were used to measure the levels of IFN‐β and CXCL10 cytokines in tumors from different treatment groups. The amount of IFN‐β in the cDZ@IP + L samples was found to be 13.84 times higher than that in the control group (Figure 6O). Similarly, CXCL10 levels increased by 3.71‐fold (Figure 6P), further indicating activation of the STING pathway within the tumor microenvironment. Additionally, compared to the control group, nanoagonist treatment under NIR irradiation induced varying degrees of cGAMP upregulation (Figure S39). This strongly supports that the cDZ@IP + L group, under combined treatment, generates a large amount of endogenous dsDNA, and the released Zn^2^^+^ enhances the sensitivity of cGAS, thereby producing more cGAMP. These results provide compelling evidence that the engineered cDZ@IP nanoagonist can effectively activate the STING pathway in vivo and trigger a potent antitumor immune response.

To verify whether the tumor immune microenvironment was improved, we analyzed immune cells in both tumor tissues and spleens using flow cytometry and immunofluorescence staining. As shown in Figure 6J,K, the percentage of tumor‐infiltrating CD4^+^ T cells was significantly increased in the cDZ@IP + L treatment group. Notably, compared to the PBS (5.77%), cDZ (8.34%), cDZ + L (10.8%), and cDZ@P + L (14.1%) groups, the cDZ@IP + L group exhibited the highest activation of CD8^+^ T cells (20.3%, Figure 6L,M and Figure S40). Immunofluorescence staining of CD8^+^ T cells in tumor tissues further supported the flow cytometry results (Figure S41). Additionally, the proportion of immune cells in the spleen was assessed. The proliferative CD4^+^ and CD8^+^ T cell populations were found to be 4.84‐fold and 3.4‐fold higher in the cDZ@P + L group compared to the control, with cell frequencies reaching 16.9% and 19.7%, respectively (Figure S37C‐F). Notably, significant increases in the secretion of IL‐6 (Figure 6Q) and TNF‐α (Figure 6R) were observed in tumors treated with cDZ@P + L. These data collectively demonstrate that the cDZ@IP nanoagonist effectively enhances cGAS‐STING activation and boosts tumor immune stimulation in vivo.

H&E staining of major organs, including the heart, liver, spleen, lungs, and kidneys, following cDZ@IP treatment showed no signs of toxicity (Figure S42). Additionally, blood biochemical parameters, including ALP, ALT, AST, GGT, BUN, and CREA, as well as hematological indices, such as WBC, Lymph, MON, NEU, PLT, and RBC, remained unchanged across different treatment groups (Figure S43). The hemolysis assay results were also within the normal range (Figure S44). These findings indicate that cDZ@IP nanoagonists exhibit excellent biosafety and biocompatibility.

### 2.7 Immune Therapeutic Mechanisms of cDZ@IP Through STING Pathway Activation

**FIGURE 7:**
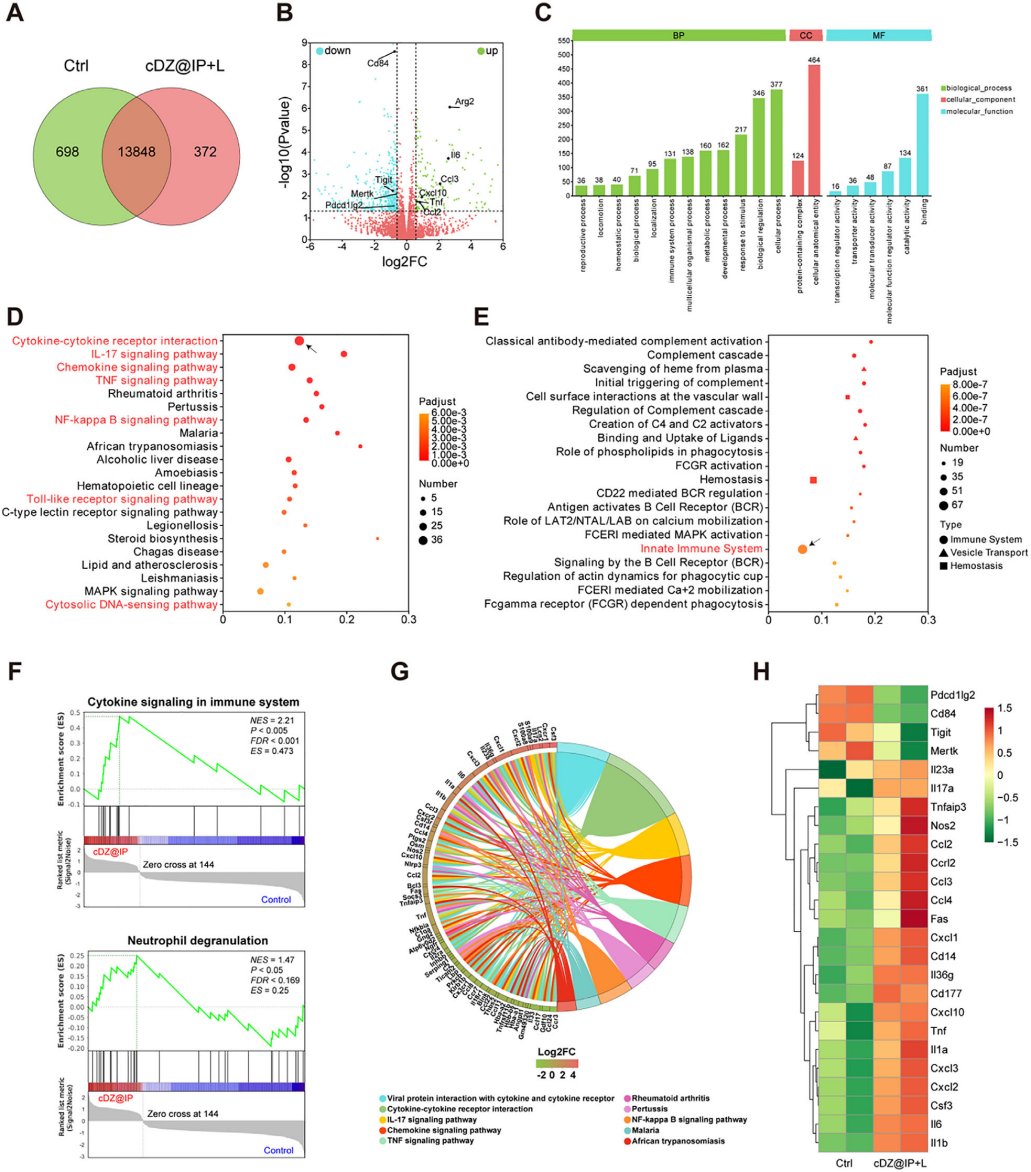
Transcriptomic analysis of tumor tissues after cDZ@IP treatment. (A) Venn diagram showing the number of shared and unique genes between Control and cDZ@IP + L‐treated tumor samples; (B) Volcano plot of DEGs between the Control group and cDZ@IP + L group (The significance threshold in the volcano plots was set at *p* < 0.05 and |log2 FC| > 1.5. p‐values were adjusted for multiple hypothesis testing and calculated using a negative binomial distribution and the significance test using a two‐sided probability); (C) GO term analysis of differential biological process profiles based on RNAseq after cDZ@IP + L treatment (The enrichment analysis of DEGs in GO used an overenrichment analysis algorithm, with p‐values based on a hypergeometric test); (D) KEGG term analysis of differential metabolic pathway based on RNAseq after the cDZ@IP + L treatment (the enrichment analysis of DEGs in KEGG used an overenrichment analysis algorithm, with p‐values based on a hypergeometric test); (E) Reactome pathway enrichment analysis of DEGs in tumor tissues after cDZ@IP + L treatment (the enrichment analysis used an overrepresentation analysis algorithm, and p‐values were calculated using a hypergeometric test); (F) GSEA enrichment plots of DEGs in the cDZ@IP + L group; (G) Pathway–gene network map showing the association between key DEGs and enriched immune‐related pathways after cDZ@IP + L treatment (the width of the ribbons reflects the strength of association, and colors correspond to different signaling pathways); (H) Heat map for showing significantly up‐regulated and down‐regulated genes in MC38 tumors with the treatment of cDZ@IP + L (Gene expression values were normalized and scaled across samples, Color scale represents relative expression levels: red = upregulated, green = downregulated).

The therapeutic mechanism of cDZ@IP + L treatment in MC38 tumor‐bearing mice was evaluated through transcriptomic analysis. As shown in Figure 7A, the Venn diagram reveals significant differences in the original transcripts between the control and cDZ@IP + L groups. The heatmap of the differential genes is shown in Figure S45. Compared to the control group, 151 upregulated genes and 438 downregulated genes were identified in the tumors treated with cDZ@IP + L (Figure 7B). Among the upregulated genes, key factors such as IL6 and CXCL10 exhibited notable changes. To further investigate the biological and metabolic pathways associated with these differentially expressed genes (DEGs) following cDZ@IP + L treatment, Kyoto Encyclopedia of Genes and Genomes (KEGG) pathway and Gene Ontology (GO) analyses were performed. GO analysis revealed that most DEGs were enriched in immune‐related pathway categories (Figure 7C), such as immune system processes and biological regulation, indicating a strong correlation between cDZ@IP + L treatment and immune‐related pathways. KEGG pathway analysis of the differential genes also highlighted their association with major STING activation pathways, including Cytokine‐cytokine receptor interaction, Chemokine signaling pathway, Cytosolic DNA‐sensing pathway, and NF‐kappa B signaling pathway (Figure 7D). Additionally, Reactome enrichment analysis showed that the majority of DEGs were enriched in the innate immune system category (Figure 7E). Gene Set Enrichment Analysis (GSEA) was employed to further examine the enrichment of DEGs within gene sets, offering deeper insight into the biological mechanisms of the combined therapy. The results demonstrated that cDZ@IP + L treatment induced significant gene changes related to Cytokine signaling in immune system and Neutrophil degranulation (Figure 7F). The enrichment chord diagram in Figure 7G illustrates the increased gene expression and the associated KEGG enrichment pathways, including Cytokine‐cytokine receptor interaction, Chemokine signaling pathway, NF‐kappa B signaling pathway, and TNF signaling pathway. To confirm the specific therapeutic mechanisms of cDZ@IP + L treatment, a heatmap of the relevant DEGs was generated. cDZ@IP + L‐mediated STING pathway activation triggered a multifaceted tumor‐killing response (Figure 7H), including the induction of interferon‐stimulated genes (e.g., Nos2), pro‐inflammatory cytokines (e.g., Tnf, Il1a, Il6, Il17a, and Il23a), leukocyte‐recruiting chemokines (e.g., Ccl2, Ccl3, Ccl4, Cxcl1, Cxcl2, Cxcl3, and Cxcl10), and typical markers of T cell or dendritic cell activation (e.g., Tigit, Cd84, Pdcd1lg2, Mertk, and Ccrl2). Collectively, these findings suggest that cDZ@IP + L treatment activates the systemic STING pathway, thereby triggering a robust anti‐tumor immune response to eliminate tumors.

### 2.8 Evaluation of cDZ@IP‐Activated STING Pathway‐Mediated Systemic Antitumor Responses

**FIGURE 8:**
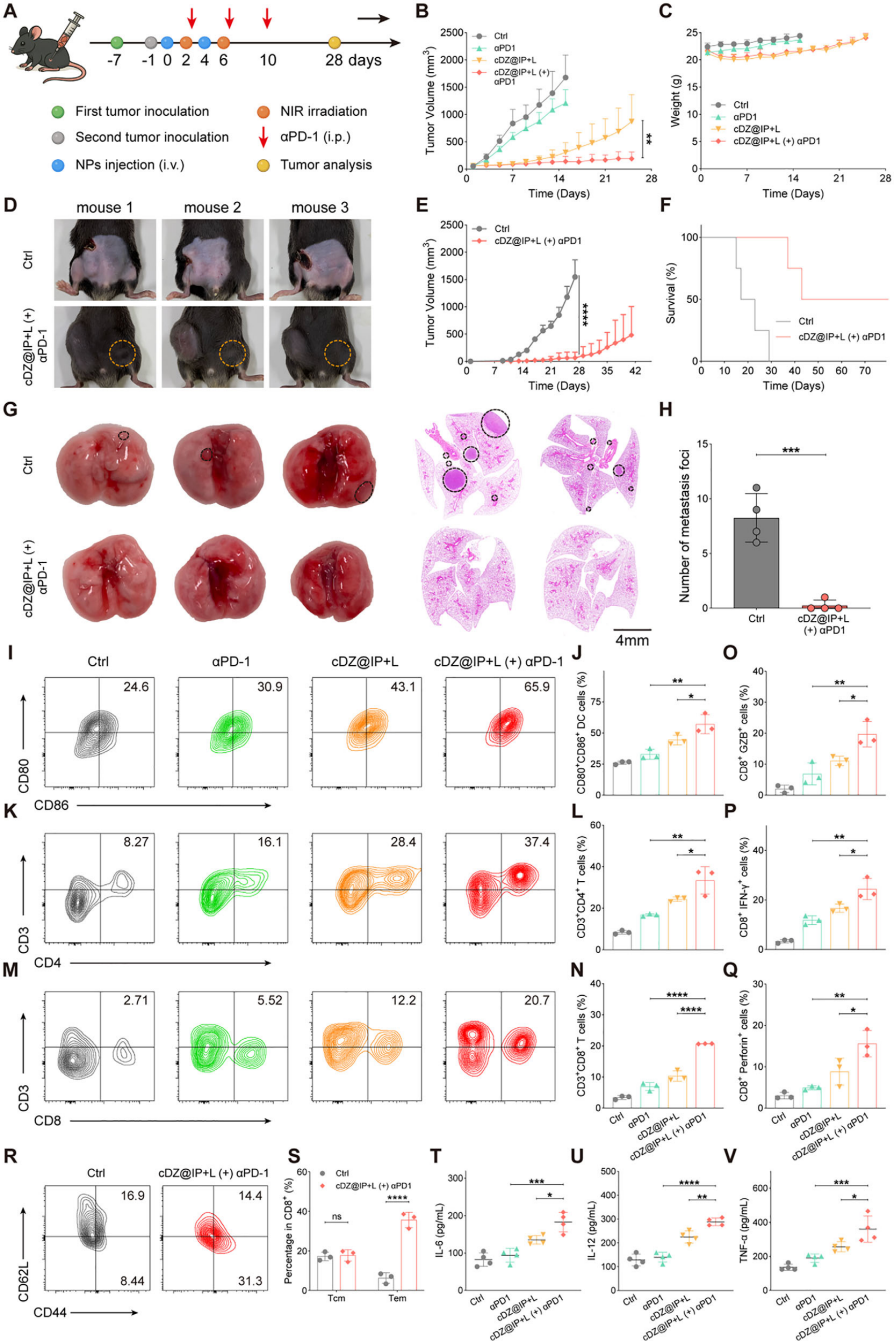
cDZ@IP combined with αPD‐1 induces systemic antitumor immunity and immune memory in bilateral MC38 tumor model. (A) Schematic illustration of cDZ@IP + L in combination with anti‐PD‐1 (αPD‐1) for inhibiting primary and secondary tumor growth; (B) Growth curves of primary tumors following various treatments (n = 4); (C) Average body weight curves of mice after varied treatments (n = 4); (D) Photographs of tumor growth on the 20th day after the inoculation of secondary tumors (yellow circle: residual tumors); (E) Growth curves of secondary tumors in each group (n = 4); (F) Survival time of the treated mice (n = 4); (G) Photographs and H&E staining of tumor nodules in lungs of naive mice and mice treated with cDZ@IP + L + αPD‐1 (black circle: metastatic nodules, scale bar: 4 mm); (H) Quantification analysis of tumor nodules in the lungs in the different treatment groups (n = 4); (I‐N) Flow cytometry analysis and quantification of matured DCs (I, J), CD4^+^ T cells (K, L), and CD8^+^ T cells (M, N) in tumors after various treatments (n = 3); (O‐Q) The percentage of GZB^+^CD8^+^ T cells (O), IFN‐γ^+^CD8^+^ T cells (P) and Perforin^+^ CD8^+^ T cells (Q) in the tumors (n = 3); (R, S) Flow cytometry analysis (R) and quantification (S) of the ratios of and CD8^+^ T memory subsets (Tcms: CD62L^+^CD44^+^; Tems: CD62L^−^CD44^+^) in tumors (n = 3); (T‐V) Detection of the levels of IL‐6 (T), IL‐12 (U), and TNF‐α (V) in serum from mice rechallenged with secondary tumors after 7 days (n = 3). Data are presented as mean ± standard deviation from a representative experiment (n ≥ 3 biologically independent samples). *P* values were determined using unpaired two‐tailed Student's *t*‐test, one‐way ANOVA with Tukey's multiple comparisons post hoc test, or two‐way ANOVA with Sidak's test, as appropriate. ****p < 0.0001, ***p < 0.001; **p < 0.01; *p < 0.05.

The promising immune response induced by cDZ@IP‐activated STING pathway encourages further evaluation of the synergistic antitumor effects of immune checkpoint blockade (αPD‐1) in the MC38 bilateral tumor model (Figure 8A). Tumor‐bearing mice were treated via intravenous administration of cDZ@IP NPs (100 µL, 5 mg kg^−1^) and intraperitoneal injection of αPD‐1 (100 µg per mouse). As clearly shown in Figure 8B, monotherapy with αPD‐1 did not induce a significant antitumor effect, leading to rapid death of tumor‐bearing mice, similar to the control group. cDZ@IP + L treatment exhibited significant antitumor effects within the first 14 days; however, tumor recurrence was observed in the primary tumors. Notably, when cDZ@IP + L was combined with αPD‐1, the primary tumors were almost completely eradicated. No abnormal weight changes were observed in mice treated with cDZ@IP + L (+) αPD‐1 (Figure 8C).

Next, we also assessed the growth of distant tumors. The results showed that distant tumors in the control group progressed rapidly. In contrast, most mice in the cDZ@IP + L (+) αPD‐1 group exhibited good tumor control (Figure 8D,E), achieving long‐term survival (Figure 8F). As shown in Figure 8G,H, numerous lung nodules were observed in the control group, whereas almost no lung metastatic nodules were detected in the cDZ@IP + L (+) αPD‐1 group. These results suggest that cDZ@IP + L (+) αPD‐1 treatment provides long‐term immune protection against tumor recurrence and metastasis.

The mature DCs in primary tumors was examined to further investigate the activation of antitumor immunity following various treatments. Notably, the DC maturation level was highest in the cDZ@IP + L (+) αPD‐1 group (65.9%), which was 2.67‐, 2.13‐, and 1.52‐fold higher than that in the control group (24.6%), αPD‐1 (30.9%), and cDZ@IP + L (43.1%), respectively (Figure 8I,J and Figure S46). Additionally, the percentage of tumor‐infiltrating CD4/CD8 T cells in the primary tumors was ∼37.4%/20.7% in the cDZ@IP + L (+) αPD‐1 group (Figure 8K‐N and Figure S47), the highest among all treatment groups. Specifically, the recruitment of CD4/CD8 T cells to the tumors after cDZ@IP + L (+) αPD‐1 treatment was ∼4.52/7.63, ∼2.32/3.75, and ∼1.31/1.69 times higher than in the control group, αPD‐1 group, and cDZ@IP + L group, respectively. Moreover, tumors treated with cDZ@IP + L (+) αPD‐1 exhibited a significant increase in GZB^+^CD8^+^, IFN‐γ^+^CD8^+^, and Perforin^+^CD8^+^ T cells (Figure 8O–Q, and Figures S48 and S49). These findings suggest that the combination of cDZ@IP + L and αPD‐1 could more effectively enhance the superior systemic immune responses for directly killing tumor cells.

Since cDZ@IP + L (+) αPD‐1 triggered a robust systemic immune response, effector memory T cells (Tems, CD62L^−^CD44^+^) and central memory T cells (Tcms, CD62L^+^CD44^+^) in the tumors were further examined to assess the immune memory response. Interestingly, compared to the control group, the percentage of Tem cells in CD8 T cells was significantly increased after cDZ@IP + L (+) αPD‐1 treatment (Figure 8R,S and Figure S50). A similar result was observed in CD4 T cells (Figure S51). As expected, mice treated with cDZ@IP + L (+) αPD‐1 exhibited significantly elevated levels of key antitumor cytokines, including IL‐6, IL‐12, and TNF‐α (Figure 8T–V). These findings suggest that STING pathway activation by cDZ@IP + L, in combination with immune checkpoint blockade, elicits a more potent systemic antitumor immune response.

## 3 Conclusion

In this work, we successfully developed cDZ@IP NPs, a multifunctional nanoagonist with dual responsiveness to endogenous tumor microenvironment cues and exogenous mild photothermal stimulation. These nanoparticles exhibit precise tumor‐targeting capability and degrade selectively under tumor‐specific conditions, leading to elevated ROS levels and mitochondrial stress, which in turn release large amounts of mtDNA into the cytoplasm. Simultaneously, the controlled release of the chemotherapeutic agent TPT, administered at only 1/16 of the standard dose, further enhances DNA damage and promotes cytoplasmic dsDNA accumulation, serving as an effective trigger for cGAS‐STING activation within the designed nanoplatform [63]. Notably, the concentration of TPT used in this study was determined based on its TPT‐equivalent dose within the nanoparticle formulation to ensure a direct and fair comparison between free TPT and nanoparticle‐loaded TPT. As a clinically approved topoisomerase I inhibitor, TPT provides translational relevance, and the nanoplatform is designed to enhance tumor‐localized delivery rather than mimic systemic plasma concentrations. This synergistic mechanism specifically activates the cGAS‐STING pathway, with Zn^2^^+^ coordination enhancing DNA sensing, ultimately inducing a potent systemic antitumor immune response.

Beyond immune activation, cDZ@IP NPs possess mild photothermal properties that facilitate localized tumor ablation and accelerate nanoparticle degradation. This further enhances ICD and promotes the release of tumor antigens, contributing to strong type I interferon responses and effective antigen‐specific T cell activation. Notably, this immune activation not only suppresses primary tumors but also inhibits metastasis and promotes long‐term immune memory. Moreover, the therapeutic efficacy of cDZ@IP NPs is significantly amplified when combined with immune checkpoint blockade (αPD‐1), achieving synergistic and durable tumor suppression. Importantly, this strategy contributes to the reprogramming of the tumor immune microenvironment, facilitating the conversion of immunologically “cold” tumors into “hot” tumors with enhanced immune infiltration and responsiveness.

These findings suggest that rationally designed immune‐responsive nanoagonists can bridge the gap between targeted chemotherapy and immunotherapy. The integration of immune activation, precise delivery, and photothermal assistance in a single system represents a promising strategy to overcome current limitations of conventional cancer treatments. Building upon this concept, our proposed nano‐metabolomics–enabled immune activation strategy will guide the rational design of therapeutic metabolites and establish a new paradigm bridging nanomedicine and cancer immunotherapy.

From a mechanistic perspective, it is worth noting that TPT is not the only chemotherapeutic agent capable of inducing DNA damage‐mediated cGAS‐STING activation. Other clinically used drugs, including anthracyclines, platinum‐based agents, and PARP inhibitors, have also been reported to trigger similar immunostimulatory effects through cytosolic DNA accumulation or micronucleus formation. However, TPT was selected in this study because, as a topoisomerase I inhibitor, it induces replication‐associated DNA damage and promotes micronucleus formation, which are key upstream events for efficient cGAS‐STING activation. More importantly, the integration of TPT into our TME‐responsive nanoplatform enables controlled release and synergistic amplification with ROS generation and NIR‐triggered effects, thereby enhancing immunostimulatory efficacy. In addition, as a clinically approved drug, TPT offers favorable translational potential compared to non‐approved agents.

However, some limitations should be acknowledged. Although encouraging antitumor responses were observed in preclinical models, the biosafety, pharmacokinetics, and immune variability across different tumor types and patient immune statuses require further investigation. Additionally, while Zn^2^^+^ plays a key role in STING activation, its systemic effects and potential off‐target immune modulation warrant closer scrutiny in future studies.

In summary, cDZ@IP NPs offer a novel and powerful approach for systemic cancer immunotherapy, combining controlled drug release, immune modulation, and photothermal enhancement in a single agonist. This strategy holds strong translational potential for treating primary and metastatic tumors and provides a versatile framework for the development of next‐generation immune nanomedicine.

## 4 Experimental Section

### 4.1 Ethical Regulations

All research complied with all relevant ethical regulations. All animal procedures were conducted strictly with institutional guidelines and were approved by the Medical Ethical Board of the Sir Run Run Shaw Hospital, School of Medicine, Zhejiang University (SRRSH202402313). The maximal tumor burden permitted by the Ethics Committee of Run Run Shaw Hospital affiliated with Zhejiang University is 2000 mm^3^. All the tumor burden in our experiment did not exceed this criterion. At the end of the mouse experiments, mice were euthanized according to animal welfare standards (euthanasia of all animals was performed using isoflurane in small animal anesthetics).

### 4.2 Materials

Zinc acetate dihydrate (≥99.0% (wt/wt); Sigma–Aldrich, cat. no. 96459). 2‐Methylimidazole (99% (wt/wt); Sigma–Aldrich, cat. no. M50850). Dopamine hydrochloride (Sigma–Aldrich, cat. no. H8502). Topotecan hydrochloride (TPT) were purchased from MedChemExpress (Shanghai, China). Indocyanine green (ICG) were purchased from Absin (Shanghai, China). NH_2_‐PEG_2k_, cRGD_fk_‐PEG_2k_‐NH_2_ were purchased from ruixi Biological Technology (Xi'an, China). Tris were purchased from Yien (Shanghai, China). 5,5′‐dithiobis (2‐ nitrobenzoic acid) (DTNB) were purchased from Aladdin (Shanghai, China). Cell counting kit‐8 (CCK‐8) were purchased from APExBIO (U.S.). The Mito‐Tracker Red CMXRos, the Lyso‐Tracker Red, the Calcein/PI Cell Viability/Cytotoxicity Assay Kit, the Reactive Oxygen Species Assay Kit, the DNA Damage Assay Kit by γ‐H2AX Immunofluorescence, and the Mitochondrial Membrane Potential Assay Kit with JC‐1 were purchased from Beyotime Biotechnology (Shanghai, China). The CheKine Micro Reduced Glutathione (GSH) Assay Kit were purchased from Abbkine (Wuhan, China). The Zinquin ethyl ester was purchased from MedChemExpress (Shanghai, China). Annexin V‐FITC Apoptosis Detection Kit was purchased from Liankebio (Hangzhou, China). DAPI, Triton X‐100, and Seal with goat serum were purchased from Solarbio (China). The PicoGreen dsDNA Assay Kit was purchased from Maokang Biotechnology Co., Ltd (Shanghai, China). Fluorescein (FITC) Tunel Cell Apoptosis Detection Kit and Hematoxylin & Eosin dye were purchased from Wuhan Sevicebio Technology Co., Ltd. (China). Collagenase Type IV and DNase I were purchased from Sigma (USA). ATP content determination kits were purchased from APPLYGEN (Beijing, China). Mouse IL‐4 Recombinant Protein and Recombinant Murine GM‐SCF were purchased from PeproTech (USA). BD Cytofix/Cytoperm Kit (BD, Cat No. 554714) and Leukocyte Activation Cocktail (BD, Cat No. 550583) were purchased from BD bioscience (USA). The tumor necrosis factor alpha (TNF‐α), interferon beta (IFN‐β), CXCchemokineligand‐10 (CXCL10), and interleukin 6 (IL‐6) ELISA Kit were purchased from Jianglai Industrial Co., Ltd (Shanghai, China). The antibodies used for western blotting, immunofluorescence staining, and flow cytometry were provided in Table S1. Deionized water obtained from the Millipore system (Direct‐Q 5, FRA) was used in all preparations.

### 4.3 Characterization

Transmission electron microscopy (TEM, HT‐7700, HITACHI, Japan) and field‐emission scanning electron microscope (SEM, SU8010, HITACHI, Japan) were used to reveal the morphology of the nanoparticles, providing detailed insights into their structural features. Elemental mapping and high‐angle annular dark‐field scanning transmission electron microscope (HADDF‐STEM) images were performed on Tecnai G2 F20 transmission electron microscope. The zeta potential and hydrodynamic size distribution of the nanoparticles were determined by dynamic light scattering (DLS, NanoZS, Malvern, U.K.). Structural analysis was carried out using x‐ray diffraction (XRD) patterns, obtained with a Rigaku x‐ray diffractometer (XRD‐6000, Shimadzu, Japan) utilizing Cu Kα radiation (λ = 1.5406 Å). Fourier transform infrared spectroscopy was obtained from a Vertex 70 Fourier transform infrared spectrometer (FTIR, Bruker, Germany). To confirm the surface composition of nanoparticles, x‐ray photoelectron spectroscopy (XPS, Thermo Scientific, USA) was employed. The entrapment efficiency of ICG was investigated by ultraviolet‐visible (UV−vis) spectroscopy (UV‐2600, Shimadzu, Japan). The concentrations of the chemotherapeutic agent and Zn^2+^ were quantitatively determined using by the HPLC (Agilent1100, USA) and ICP‐MS (NexION 300XX, PerknElmer, USA), respectively. Additionally, confocal laser scanning fluorescence microscopy (CLSM, TCS SP8, Leica, Germany) was employed to capture high‐resolution fluorescence images. Flow cytometry analyses were performed using a CytoFLEX LX flow cytometer (Beckman Coulter, CA, USA). Cell viability was assessed using a microplate reader (Invitrogen, Carlsbad, CA). Western blot (WB) signals were captured using a Tanon 5200 imaging system (Tanon, Shanghai, China). In vivo biodistribution fluorescence images were obtained IVIS Spectrum system (PerkinElmer).

### 4.4 Synthesis of ZIF8@ICG/TPT Nanoparticles (Z@IP NPs)

Z@IP nanoparticles (NPs) were synthesized via a facile one‐pot method. Briefly, 0.374 g of zinc acetate dihydrate and 1.12 g of 2‐methylimidazole were each dissolved in 5 mL of ultrapure water. Subsequently, 5 mg of TPT and 5, 10, or 15 mg of ICG were added to the 2‐methylimidazole solution and stirred thoroughly to ensure complete dissolution and uniform mixing. The zinc acetate solution was then added dropwise to the mixture under continuous stirring at 500 rpm for 1 h. The reaction mixture was allowed to age at room temperature for 16 h. The resulting nanoparticles were collected by centrifugation at 10 000 rpm for 10 min and washed three times with deionized water. Finally, the purified nanoparticles were re‐dispersed in ultrapure water for subsequent experiments.

### 4.5 Synthesis of cRGD‐PDA/ZIF8@ICG/TPT Nanoparticles (cDZ@IP NPs)

Z@IP nanoparticles (NPs) were functionalized with cRGD_fk_‐PEG_2k_‐NH_2_ via a PDA‐mediated surface modification strategy. Briefly, 25 mg of Z@IP NPs were dispersed in 50 mL of Tris‐HCl buffer, followed by the addition of 5 mg of dopamine hydrochloride, which was dissolved directly into the suspension. The reaction mixture was stirred at room temperature for 4 h to allow the formation of a PDA coating. The resulting nanoparticles were then collected by centrifugation at 10 000 × *g* for 10 min and washed three times with ultrapure water. The PDA‐coated nanoparticles were subsequently re‐dispersed in 20 mL of phosphate buffer (Na_2_HPO_4_/NaH_2_PO_4_, pH 8.5), and 20 mg of cRGD_fk_‐PEG_2k_‐NH_2_ was added. The mixture was stirred overnight at 4°C in the dark to facilitate ligand conjugation. The final product was purified by centrifugation (10 000 × *g*, 10 min) and washed three times with ultrapure water for further use. As a control, nanoparticles modified with an equal mass of NH_2_‐PEG_2k_ were prepared using the same procedure, and designated as nDZ@IP.

Similarly, cDZ (cRGD_fk_‐PEG_2k_‐NH_2_–PDA/ZIF8) and cDZ@P (cRGD_fk_‐PEG_2k_‐NH_2_–PDA/ZIF8@TPT) were synthesized following the same protocol, with the exclusion of TPT or ICG from the precursor solution, respectively.

### 4.6 GSH Depletion Measurement

cDZ@IP NPs concentration‐dependent GSH depletion. GSH solution (final concentration 3 mm) was fully mixed with cDZ@IP NPs solution with different concentrations (0–20 µg mL^−1^) for 6 h, and 2 mL of the above‐mixed solution was centrifuged. The supernatant was collected, then DTNB (10 µL, 10 mg mL^−1^) was added to the above‐collected supernatant. The absorbance curves at different concentrations were recorded by UV–vis spectrophotometer after incubation for 10 min.

Time‐dependent GSH depletion. GSH solution (final concentration 3 mm) was thoroughly mixed with cDZ@IP NPs solution (final concentration 20 µg mL^−1^) at different times. 2 mL of the above‐mixed solution was centrifuged at 1, 2, 4, 8, and 24 h, respectively, and the supernatant was collected, and DTNB (10 µL, 10 mg mL^−1^) was added to it for 10 min. The absorbance curves of different time points were recorded by UV–vis spectrophotometer.

### 4.7 TPT Release

TPT release profiling was conducted following previously reported protocols. cDZ@IP NPs were dispersed in various buffer solutions, including pH 7.4, pH 5.8, pH 5.8 + 10 mm GSH, and pH 5.8 + 10 mm GSH + laser. The suspensions were incubated at 37°C with shaking at 120 rpm for 48 h. At designated time points, 3 mL of the external release medium was collected and replaced with an equal volume of fresh buffer. The amount of TPT released into the medium was quantified by HPLC.

### 4.8 Zn^2+^ Release

cDZ@IP nanoparticles were incubated under various conditions. The concentration of Zn^2^^+^ released into the supernatant was quantified using ICP‐MS (NexION 300XX, PerkinElmer, USA). Meanwhile, the morphological changes of the nanoparticles after incubation were observed by TEM.

### 4.9 Measurement of ^1^O_2_ Generation

The ^1^O_2_ generation from NIR‐activated cDZ@IP was detected by Singlet Oxygen Sensor Green (SOSG). Typically, SOSG (5 µm) was added to dispersions of cDZ@IP (final concentration: 20 µg mL^−1^) under various conditions, including pH 7.4, pH 5.8, 10 mm glutathione (GSH), and pH 5.8 with 10 mm GSH. The samples were then irradiated with an 808 nm laser at a power density of 1.0 W cm^−2^ for 5 min. The fluorescence intensity of SOSG at Ex/Em = 504/525 nm was then detected. As controls, the ^1^O_2_ generation in the presence of SOSG in PBS with laser irradiation was also tested for comparison. In addition, the concentration‐dependent, time‐dependent, and laser power‐dependent ^1^O_2_ generation of cDZ@IP was also investigated.

### 4.10 In Vitro Photothermal Effects

cDZ@IP NPs concentration‐dependent photothermal performance. Specifically, cDZ@IP dispersions at varying concentrations (0–80 µg mL^−1^) were irradiated with an 808 nm NIR laser at a constant power density of 1.0 W cm^−2^ for 5 min. The temperature of the solution was monitored by an FLIR E50 Infrared (IR) camera (FLIR Systems Inc., Wilsonville, OR, USA).

Laser power‐dependent photothermal performance. Typically, 400 µL of cDZ@IP dispersion (final concentration: 20 µg mL^−1^) was irradiated with an 808 nm NIR laser at varying power densities (0.5–2.0 W cm^−2^) for 5 min. The temperature change was monitored during irradiation. PBS was used as a negative control in this experiment.

To evaluate the photothermal stability of cDZ@IP NPs, heating and cooling were repeated for five cycles.

To measure the photothermal conversion efficiency (*η*), cDZ@IP NPs (final concentration: 20 µg mL^−1^) were exposed to 808 nm irradiation at a density of 1.0 W cm^−2^ for 15 min to reach thermal equilibrium and then the solution was cooled to room temperature. The temperature of the solution was recorded at an interval of 1 s during this process. The *η* was determined using Equation (1), gaining a deeper understanding of the photothermal conversion capability of the nanoagonist.

(1) $$\eta =\frac{hs\left(T_{max}-T_{surr}\right)-Q_{s}}{I\left(1-10^{-A_{808}}\right)}\;$$

where *h* is the heat transfer coefficient and *s* is the surface area of the container. *Q_s_* is light absorbance of the solvent, *I* is the laser energy for the 808 nm laser, and *A*_808_ is related to the absorbance value of cDZ@IP NPs tested by analysis of the UV−vis spectrum at 808 nm. The total heat transfer capacity of the system under the unit temperature difference (*hs*) was calculated using Equation (2),

(2) $$hs=\frac{mC_{water}}{\tau_{s}}\;$$

where *m* is the mass of the solution containing the NPs, *C_water_* is the specific heat capacity of the solution (*C_water_* =  4.2 J *g* ^−1^ *K* ^−1^), and τ_*S*_ is the associated time constant calculated from the linear time‐dependent data collected in the cooling period. The time correlation coefficient (τ_*S*_) was calculated using Equation (3),

(3) $$t=-\tau_{S}\ln\theta$$

*θ* is a dimensionless parameter, known as the driving force temperature, and was calculated using Equation (4).

(4) $$\theta =\frac{T-T_{Surr}}{T_{max}-T_{Surr}}\;$$

where, *T_max_* and *T_Surr_* are the maximum steady state temperature and the environmental temperature, respectively.

### 4.11 Cell Culture

Mouse CRC cells (MC38) and HUVECs, were cultured in DMEM medium containing 10% FBS and 1% antibiotics (penicillin‐streptomycin, 10 U mL^−1^). Bone marrow‐derived dendritic cells (BMDCs) were generated from murine bones according to an established method. Briefly, bone marrow was extracted from isolated femurs and tibias. The samples were then incubated with eBioscience 1 × RBC lysis buffer for 3 min to lysered blood cells and centrifuged at 300 × *g* for 5 min to collect the remaining cells. The collected cells were dispersed into RPMI 1640 medium supplemented with 10% FBS, 100 µg mL^−1^ penicillin/streptomycin, 2 mm L‐glutamine, 0.05 mm β‐ME, 20 ng mL^−1^ GM‐CSF and 10 ng mL^−1^ IL‐4. Fresh medium was provided every 2 days. After 7 days of culture, the non‐adherent cells were collected for further investigation. All the cells were incubated at 37°C and 5% carbon dioxide (CO_2_).

### 4.12 Animals

The 6‐week‐old healthy male C57BL/6J black mice (approximately 20 g in body weight) were purchased from Charles River Laboratories. and kept in the pathogen‐free animal room. The animals were kept in ventilated cages and exposed to light for around 10 h. The ambient temperature was controlled at 22°C, and the humidity was controlled between 50% and 60%.

### 4.13 In Vitro Cellular Uptake of cDZ@IP NPs

To study the cell endocytosis of functionalized cDZ@IP nanoagonists, MC38 cells were seeded into confocal dishes at a density of 1 × 10^5^ per dish and cultured at 37°C for 24 h. FITC‐labeled DZ@IP or cDZ@IP nanoagonists (Green) were added for another 4 h and then cells were washed three times with PBS and stained with Phalloidin and Hoechst 33342 before observing by CLSM. The cells could be collected and analyzed by flow cytometry as well.

### 4.14 Cell Viability

First, HUVECs or MC38 cells were seeded in a 96‐well plate at a density of 1 × 10^4^ cells per well were cultured for 24 h. Subsequently, different concentrations (0, 10, 20, 30, 40, 50, and 60 µg mL^−1^) of DZ@IP or Z@IP NPs in the fresh culture medium were added into each well to replace the existing cell culture media. The cells were then incubated for an additional 24 h. Following the incubation period, cell viability was assessed using the CCK‐8 assay.

### 4.15 The GSH Depletion Assay of cDZ@IP NPs in MC38 Cells

Intracellular GSH depletion caused by cDZ@IP NPs was investigated by CheKine Micro Reduced Glutathione Assay Kit. First, MC38 cells were seeded in 6‐well plates at a density of 1 × 10^5^ cells per well and incubated overnight. Then, the cells were treated with different groups (control, dopamine, cDZ@IP, TPT, ICG, and ZIF‐8) for 6 h. Subsequently, the cells of each group were collected and added with extraction buffer to freeze‐thaw. Then, the supernatant was collected by centrifuge (8000 g, 10 min) at 4°C. Next, deionized water (20 µL), standard solution (0, 3.125, 6.25, 12.5, 25, 50, 100, and 200 µg mL^−1^, 20 µL), and supernates (20 µL) of each group were added to 96‐well plate and mixed with assay buffer (140 µL) and chromogen (40 µL) for 2 min at room temperature. Lastly, the absorption at 412 nm was detected by microplate reader and the concentration of GSH in mixture could be calculated through the standard concentration curve.

### 4.16 Intracellular Zn^2+^ Content

MC38 cells were seeded into a six‐well plate (5 ×10^5^ cells per well), cultured for 24 h, and then incubated with PBS, DZ@IP (20 µg mL^−1^), or cDZ@IP (20 µg mL^−1^) for 8 h. Then, the medicated culture medium was washed, the cells were washed thrice with PBS, and the number of cells per well was accurately counted. ICP‐MS was used to detect the level of Zn^2+^ in the cells.

### 4.17 Distribution of Intracellular Zn^2+^

MC38 cells were inoculated into confocal culture dishes (2 × 10^5^ cells per dish), cultured for 24 h, and then incubated with cDZ@IP (20 µg mL^−1^) for 0, 2, 4, or 8 h. The medicated culture medium was then washed, and the cells were washed thrice with PBS. The MitoTracker fluorescent probe was used to stain the mitochondria (120 nm, 20 min), and Zinquin ethyl ester was used to stain Zn^2+^ (5 µm, 15 min). The cells were then observed under a CLSM.

### 4.18 Detection of Intracellular ROS

MC38 cells were seeded into confocal dishes at a density of 5 × 10^4^ cells per dish and incubated at 37°C for 24 h. Then, the cells were treated by the following five groups for 8 h: Control, cDZ (20 µg mL^−1^), cDZ + L (20 µg mL^−1^), cDZ@P + L (µg mL^−1^), and cDZ@IP + L (20 µg mL^−1^). Then, the cells were washed twice with PBS after endocytosis and added with DMEM medium. The group of cDZ + L, cDZ@P + L, and cDZ@IP + L were irradiated by NIR laser (808 nm, 1.0 W cm^−2^) for 5 min. Next, cells were incubated in an incubator for 2 h and the culture media was replaced by DCFH‐DA. After that, the medium was removed and the cells were washed three times with PBS. Finally, the cells were observed by CLSM to evaluate the intracellular ROS level. The above operation was repeated, and the amount of ROS was investigated by flow cytometry.

### 4.19 Detection of Mitochondrial Membrane Potential

JC‐1 is a cationic dye that accumulates in the mitochondrial matrix in response to high mitochondrial membrane potential (Δψm), forming J‐aggregates that emit red fluorescence. In contrast, under conditions of low Δψm, JC‐1 remains in its monomeric form and emits green fluorescence. MC38 cells were seeded in confocal dishes at a density of 5 × 10^4^ cells per dish and incubated at 37°C for 24 h. The cells were then treated with one of the following five groups for 8 h: Control, cDZ (20 µg mL^−1^), cDZ + laser (20 µg mL^−1^), cDZ@P + laser (20 µg mL^−1^), and cDZ@IP + laser (20 µg mL^−1^). After treatment, cells were incubated with JC‐1 working solution at 37°C for 20 min. The cells were subsequently washed with JC‐1 staining buffer and immediately subjected to analysis. Fluorescence signals of JC‐1 monomers (green) were detected at Ex/Em = 514/529 nm, while signals of J‐aggregates (red) were detected at Ex/Em = 585/590 nm. The assay was repeated in parallel, and changes in mitochondrial membrane potential were further quantified by flow cytometry.

### 4.20 The Therapeutic Efficacy of cDZ@IP NPs in MC38 Cells

MC38 cells were transfected with cDZ@IP NPs (0–40 µg mL^−1^) for 12 h, and then subjected to 5 min NIR irradiation (with a power density of 1.0 W cm^−2^) or left without irradiation. As control groups, cancer cells treated with PBS and 5 min NIR irradiation were employed. Subsequently, the cell survival rate was assessed using the CCK‐8 method.

### 4.21 Live/Dead Cell Co‐Staining Assay

MC38 cells were seeded into confocal dishes at a density of 5 × 10^4^ cells per dish and incubated at 37°C for 24 h. Then, the cells were treated by the following five groups for 8 h: Control, cDZ (20 µg mL^−1^), cDZ + L (20 µg mL^−1^), cDZ@P + L (20 µg mL^−1^), and cDZ@IP + L (20 µg mL^−1^). Subsequently, the cells were washed three times with PBS, irradiated (808 nm, 1.0 W cm^−2^) for 5 min, and further incubated for another 2 h. After that, the cells were co‐incubated with both Calcein AM (2 × 10^−6^ m) and propidium iodide (PI, 4 × 10^−6^ m) for 30 min in a humidified atmosphere containing 5% CO_2_ at 37°C. The cells were washed three times with PBS and observed by CLSM.

### 4.22 Cellular Apoptosis Assay

MC38 cells were seeded in 12‐well culture plates at a density of 3 × 10^4^ cells per well and incubated overnight. Then, the cells were treated by the following five groups for 8 h: Control, cDZ (20 µg mL^−1^), cDZ + L (20 µg mL^−1^), cDZ@P + L (20 µg mL^−1^), and cDZ@IP + L (20 µg mL^−1^). Then, the cells were washed three with PBS and added with DMEM medium. The group of cDZ + L, cDZ@P + L, and cDZ@IP + L were irradiated by NIR laser (808 nm, 1.0 W cm^−2^) for 5 min. Next, cells were incubated in an incubator for 2 h and harvested via trypsinization. Finally, the cells were stained with PI and annexin V‐FITC. Flow cytometry was used to evaluate the cell apoptosis.

### 4.23 DNA Damage Detection of MC38 Cells In Vitro

First, we used immunofluorescence staining of γ‐H2AX to detect DNA damage. MC38 cells were seeded into a confocal cell culture dish at a density of 5 × 10^4^ cells per well and incubated overnight. Then, the cells were treated by the following five groups for 8 h: Control, cDZ (20 µg mL^−1^), cDZ + L (20 µg mL^−1^), cDZ@P + L (20 µg mL^−1^), and cDZ@IP + L (20 µg mL^−1^). Then, the cells were washed twice with PBS and added with DMEM medium. The group of cDZ + L, cDZ@P + L, and cDZ@IP + L were irradiated by NIR laser (808 nm, 1.0 W cm^−2^) for 5 min. Next, cells were incubated in an incubator for 2 h. After that, the medium was removed and the cells were washed three times with PBS and fixed with 4% paraformaldehyde. Then, cells were treated with 0.2% Triton X‐100 for 10 min and sealed with 10% goat serum at room temperature for 1 h. Subsequently, cells were incubated with γ‐H2AX primary antibody and Fluor 488‐conjugated antibody. Finally, the cells were observed by CLSM to observe DNA damage level.

Second, the PicoGreen dsDNA Assay Kit was used to detect the dsDNA content in the cytoplasm. MC38 cells were treated with various groups (Control, cDZ, cDZ + L, cDZ@P + L, and cDZ@IP + L for 8 h. Then, the cells were washed twice with PBS and added with DMEM medium. The group of cDZ + L, cDZ@P + L, and cDZ@IP + L were irradiated by NIR laser (808 nm, 1.0 W cm^−2^) for 5 min. Next, cells were incubated in an incubator for 2 h. Subsequently, the cells of each group were collected and added with extraction buffer to freeze‐thaw. Then, the supernatant was collected by centrifuge (8000 rpm, 15 min) at 4°C and diluted 100 times with TE solution to form sample solution. The fluorescence value of dsDNA with different concentrations (12.5, 25, 50, 100, and 200 ng mL^−1^) and reactant were detected at 480/520 nm and the standard concentration curve was calculated. Finally, the sample solution (100 µL) and reactant (100 µL) were together added into a 96 well plate for 5 min, and the fluorescence value was recorded at 480/520 nm. The concentration of dsDNA in different groups could be calculated through the standard concentration curve.

### 4.24 Western Blot Assay

5 × 10^5^ MC38 cells were seeded into 6‐well plates and cultured at 37°C for 24 h. After different treatments, cells were lysed with radio immunoprecipitation assay (RIPA) lysis buffer containing 1% phenylmethanesulfonylfluoride (PMSF). Protein concentration of each group was measured by the BCA method. 10 µg proteins were electrophoresed on SDS‐PAGE and transferred onto a polyvinylidene fluoride (PVDF) membrane. Then, the membrane was blocked by 5% skim milk for 2 h, washed with Tris‐buffered saline containing Tween 20 (TBST) and incubated with certain primary antibodies overnight at 4°C. The corresponding secondary antibodies were applied for 1 h in room temperature after washing with TBST. The images of western blotting were obtained using an image analysis system.

### 4.25 cDZ@IP‐Induced ICD of MC38 Cells Under NIR Irradiations in Vitro

cDZ@IP‐induced ICDs were identified by in vitro detection of calreticulin (CRT) transposition, HMGB1 release, and ATP release. MC38 cells were seeded into a confocal cell culture dish at a density of 5 × 10^4^ cells per well and incubated overnight. Then, the cells were treated by the following five groups for 8 h: Control, cDZ, cDZ + L, cDZ@P + L, and cDZ@IP + L. Then, the cells were washed twice with PBS and added with DMEM medium. The group of cDZ + L, cDZ@P + L, and cDZ@IP + L were irradiated by NIR laser (808 nm, 1.0 W cm^−2^) for 5 min. Next, cells were incubated in an incubator for 2 h. After that, the medium was removed and the cells were washed three times with PBS and fixed with 4% paraformaldehyde. Then, cells were treated with 0.2% TritonX‐100 for 15 min (except for CRT immunofluorescence staining) and sealed with 10% goat serum at room temperature for 1 h. Subsequently, cells were incubated with CRT, and HMGB1 primary antibody and secondary antibody. Finally, the cells were observed by CLSM.

The ATP release was detected by the ATP kit. In simple terms, MC38 cells at a density of 10^5^ per well were seeded in a 6‐well plate overnight and allowed to grow until confluence. After that, cDZ@IP (20 µg mL^−1^) diluted with DMEM were added into each well and co‐cultured for 6 h. Subsequently, NIR irradiation was conducted on the cells. Two hours later, the supernatant was collected directly as the sample for detection. According to the instructions of the ATP detection kit (APPLYGEN, Beijing, China), a standard solution and ATP detection working solution were prepared, and the ATP content in the supernatant of the samples was calculated using the standard curve drawn by the standard solution according to the results obtained from the microplate reader.

### 4.26 In Vitro BMDCs Maturation Assay

Bone‐marrow derived dendritic cells (BMDCs) and MC38 cells were seeded into a transwell plate (MC38 cells in the upper chamber, BMDCs in the lower chamber) and incubated overnight. Then, the MC38 cells were treated by the following five groups for 8 h: Control, cDZ, cDZ + L, cDZ@P + L, and cDZ@IP + L. Next, the cells were washed twice with PBS and added with DMEM medium. The group of cDZ + L, cDZ@P + L, and cDZ@IP + L were irradiated by NIR laser (808 nm, 1.0 W cm^−2^) for 5 min. Subsequently, MC38 cells and BMDCs were co‐incubated. After 24 h of co‐incubation, BMDCs were collected and stained with anti‐CD11c PE, anti‐CD80 FITC, anti‐CD86 APC. FCM was used to analyze the ratio of CD11C^+^CD80^+^CD86^+^ DCs.

The proinflammatory cytokines (i.e., IL‐6 and TNF‐α) from BMDCs suspension and cGAS‐STING signal pathway factor (i.e., CXCL10, and IFN‐β) from MC38 cells suspension were detected by enzyme‐linked immunosorbent assay (ELISA) kits following a standard protocol.

### 4.27 Biodistribution

The tumor‐bearing mice were intravenously injected with Cy7.5 labeled cDZ@IP (cDZ@IP@Cy7.5). After injection, biodistribution images were collected by in vivo Imaging System (Spectrum CT, PerkinElmer, Ex/Em = 745 nm/840 nm) at preinjection and 2, 4, 12, 24, 36, and 48 h postinjection, respectively. For the ex vivo biodistribution study, mice were sacrificed at 48 h post‐injection. *Ex vivo* imaging of organs including heart, liver, spleen, lung, kidney, and tumor were collected and quantitative analyses using IVIS Spectrum imaging system.

### 4.28 In Vivo Assessments of Synergistic Anti‐Tumor Effects

Unilateral MC38 tumor‐bearing mice were randomly assigned into five groups (n = 5 per group): Control, cDZ (5 mg/kg), cDZ + L (5 mg/kg), cDZ@P + L (5 mg/kg), and cDZ@IP + L (5 mg/kg). Mice received intravenous injections of either physiological saline (Control) or the corresponding nanoparticle formulations (cDZ, cDZ@P, or cDZ@IP) on days 0 and 4. For the groups receiving laser treatment (cDZ + L, cDZ@P + L, and cDZ@IP + L), tumors were irradiated with an 808 nm laser (1.0 W cm^−2^, 5 min) at 24 h post‐injection. Tumor surface temperatures were monitored using an infrared thermal imaging system and maintained at approximately 45°C. Throughout the treatment period, tumor volume and body weight were measured every two days in a blinded manner. Tumor volume was calculated using the formula: Volume = (length × width^2^)/2. On day 18, all mice were euthanized. Mice exhibiting severe tumor‐related symptoms (e.g., labored breathing, kyphosis) or experiencing >20% body weight loss were excluded from analysis. Tumor weights were recorded, and blood samples were collected for biochemical and hematological analyses. Moreover, the main organs were collected and fixed for H&E staining. Additionally, tumor tissues were preserved in formalin for immunofluorescence staining, including TUNEL and Ki67, to evaluate the therapeutic efficacy.

### 4.29 Immune Response Triggered by cDZ@IP NPs

To study immune activation effect of NPs in vivo, the same unilateral MC38 tumor‐bearing mice model was established (group and treatment were the same as the above experiments). After the treatments, the tumor and spleen tissues were digested into single cell suspension using digestion solution (HBSS solution, collagenase IV (1 mg mL^−1^), and DNase I (0.1 mg mL^−1^)) for 30 min at 37°C. The obtained tumor tissue homogenate was filtered through a filter (40 µm), and PBS containing fetal bovine serum (5%) was added to terminate digestion. Next, the above solution was processed into cell pellet by centrifugation (500 g, 5 min) and then exposed to red blood cell lysis. Last, cell pellets were resuspended with PBS to obtain single cell suspensions. Similarly, the spleen tissue was ground, filtered, and centrifuged to obtain cell pellets. Then, single cell suspensions of spleen were prepared by red blood cell lysis and centrifugation. Subsequently, the live/dead dye was used to these single cell suspensions from tumor or spleen for 10 min on ice, and then resuspend the cells after centrifugation. CD16/32 antibody was used to these single cell suspensions for 20 min on ice to pre‐block the non‐specific binding. After that, the cells were stained by surface antibodies, including anti‐CD45‐APC/Cyanine7, anti‐CD3‐BV605, anti‐CD4‐BV421, anti‐CD8‐R718, anti‐CD44‐PE‐Cyanine7, anti‐CD62L‐Percp‐cyanine5.5, anti‐CD11c‐PE, anti‐CD80‐FITC, anti‐CD86‐APC. For the intracellular and intranuclear staining, the cells were fixed and permeabilized by BD Cytofix/Cytoperm Kit after the surface antibody labeling. Then, these cells were further stained by intracellular antibodies of IFN‐γ‐AF647, Granzyme B‐PerCP‐eFluor710, and Perforin‐PE. The fluorescence signals were detected by flow cytometry and data were analyzed by FlowJo. Additionally, one part of the tumor was collected in formalin to make the immunofluorescence tissue sections of CRT, and HMGB1, and CD8.

### 4.30 Elisa Assessment

Each group absorbed 10 µL of suspension‐containing cells and inoculated them into 12‐well plates after tumor extraction. The suspension was diluted with RPMI‐1640 complete medium containing 10% FBS, and lonomycin was diluted to 1 µg mL^−1^ and PMA to 50 ng mL^−1^. The 12‐well plates were then incubated at 37°C overnight. On the second day, the suspension was centrifuged, and the supernatant was collected for Elisa's analysis.

### 4.31 Anti‐Tumor Efficacy of cDZ@IP NPs in Combination With Anti‐PD‐1 Therapy and Tumor Rechallenge Study

MC38 tumor‐bearing mice with an average tumor volume of approximately 50 mm^3^ were randomly divided into four groups: PBS control, anti‐PD‐1 (αPD‐1) alone, cDZ@IP + L, and cDZ@IP + L (+) αPD‐1. The PBS and cDZ@IP + L groups were treated using the same intravenous injection and laser irradiation strategy described above. For the αPD‐1 group, anti‐PD‐1 antibodies (100 µg per mouse) were administered via intraperitoneal injection on days 3, 7, and 10. In the cDZ@IP + L (+) αPD‐1 group, mice received the same treatment as the cDZ@IP + L group, along with intraperitoneal injections of anti‐PD‐1 antibodies at a dose of 100 µg per mouse on days 3, 7, and 10. Tumor volume, body weight, and survival time were monitored every two days in a blinded manner. Mice exhibiting severe symptoms of tumor burden (e.g., respiratory distress, kyphosis) or experiencing more than 20% body weight loss were excluded from further analysis. For tumor rechallenge experiments, 5 × 10^5^ MC38 cells were subcutaneously inoculated into the contralateral (left) thigh of the same mice. The growth of rechallenged tumors, body weight, and survival were similarly monitored every two days. Survival curves were plotted until the endpoint, defined as either death or tumor volume exceeding 2000 mm^3^. Meanwhile, tumor tissues were harvested for flow cytometry analysis and ELISA.

### 4.32 Establishment of a Lung Colonization Model

For lung metastatic experiments, the tumor‐bearing models were established and randomly divided into the following four groups (n = 3): Control, αPD‐1 group, cDZ@IP + L group, cDZ@IP + L (+) αPD‐1 group (treatment strategy was the same as the above experiments). After 7 days of treatment, each mouse was injected with MC38 tumor cells (100 µL, 1 × 10^6^) through the tail vein. After sacrificed, lung tissue was collected for photograph and H&E to observe the lung metastasis of mice in each group.

### 4.33 Statistical Analysis

The reported values served as averages of at least three biological replicates and represent independent biological experiments with similar results. All data are presented as mean ± standard deviation (SD) analyzed using GraphPad Prism 9.0 (GraphPad Software). Two groups were analyzed by T test, and more than two groups were calculated by One/two‐way ANOVA using the Tukey/Sidak's test. The data were considered significant, when ****p < 0.0001, ***p < 0.001; **p < 0.01; *p < 0.05; ns, no significant.

## Author Contributions

K.H. was mainly responsible for the design of materials and experimental protocols and wrote the original manuscript of this article. J.W., W.Z., and Z.W. were responsible for the verification of the biological properties of the materials. K.H., J.W., W.Z., Z.W., J.C., Y.H., X.D., Y.H., X.X., H.X., Y.W., C.W., Y.Y., and Q.M. performed experiments. K.H., X.D., X.X., and Z.W. performed data, bioinformatic, and statistical analyses. Y.Q. and K.H. revised the original draft. Z.S. provided guidance and supervision. Z.S., X.L., and K.C. provided funding support. All authors reviewed the manuscript.

## Conflicts of Interest

The authors declare no conflicts of interest.

## Supporting information




**Supporting File**: advs75959‐sup‐0001‐SuppMat.docx.

## Data Availability

The authors declare that the data supporting the findings of this study are available within the paper and its Supplemental Data files and are available from the authors on request.
