# Supplementary material for: TME/NIR Dual‐Responsive Zinc‐Based Targeted Nanoagonist for Multimodal Amplification of STING‐Mediated Cancer Immunotherapy
Source: Adv Sci (Weinh). 2026 Jun 9;13(49):e23182. doi: 10.1002/advs.202523182 (PMC13336667; doi:10.1002/advs.202523182)
Supplement: Supplementary file 1 [file ADVS-13-e23182-s001.docx]

**Supplementary Information**

*Kepeng Hu^1,3,4^, Jiawei Wang^1,3,4^, Wei Zhang^1,3,4^, Zhixuan Wu^1,3,4^, Jing Chen^1,2,3,4^, Yujing He^1,3,4^, Xiyue Duan^5^, Yaoting He^5^, Xin Xin^5^, Haoyi Xiang^1,3,4^, Yijing Wang^1,3,4,^, Chengwei Wu^5^, Yang Yi^5^, Qing Meng^1,3,4^, Yuqian Qiao^6,*^, Kui Cheng^5,*^, Xiaojun Long^1,3,4,*^and Zhangfa Song^1,2,3,4,*^*

*^1^* Department of Colorectal Surgery, Sir Run Run Shaw Hospital, School of Medicine, Zhejiang University, Hangzhou, Zhejiang, 310016, China

*^2^* School of Basic Medical Sciences and Forensic Medicine, Hangzhou Medical College, Hangzhou, Zhejiang, 310007, China

*^3^* Zhejiang Key Laboratory of Biological Treatment, Hangzhou, Zhejiang, 310016, China

*^4^* Key Laboratory of Integrated Traditional Chinese and Western Medicine Research on Anorectal Diseases of Zhejiang Province, Hangzhou, Zhejiang, 310016, China

*^5^* School of Materials Science and Engineering, State Key Laboratory of Silicon and Advanced Semiconductor Materials, Zhejiang University, Hangzhou, Zhejiang, 310058, China

*^6^* School of Materials Science and Engineering, Nankai University, Tianjin, 300350, China

*^*^*Corresponding author, E-mail: songzhangfa@zju.edu.cn

**Supplementary Figures ..................................................................... 3**

**Supplementary Tables .................................................................... 55**

**Figure S1**. The expression of cGAS, TBK1 and IRF3 in the TCGA cohort.

**Figure S2**. **(**A) UMAP visualization of single-cell transcriptomes from paired tumor (T) and adjacent normal (N) tissues of five CRC patients; (B) Proportional composition of 9 major cell types identified in normal and tumor tissues; (C) UMAP plots showing the distribution of nine major cell types in normal and tumor tissues.

**Figure S3**. Enrichment of STING-related genes (cGAS, STING, TBK1, and IRF3) in epithelial cells.

**Figure S4**. SEM image of ZIF-8 NPs.

**Figure S5**. **(**A) SEM image of Z@IP NPs; (B) the average diameter of Z@IP NPs.

**Figure S6**. UV–vis–NIR absorption spectra of ZIF-8, DZ, ICG, Z@IP, and cDZ@IP.

**Figure S7**. (A) ICG concentration quantified by UV-vis spectrum; (B) The correlation curve between the concentration of ICG and the absorbance at 778 nm.

**Figure S8**. Loading efficiency and encapsulation efficiency of ICG in Z@IP NPs with the change of input molar ratio of 2-methylimidazole, zinc acetate hexahydrate, and ICG. (A) Pictures of precipitation and supernatant of Z@IP NPs after centrifugation at the end of the reaction. The pictures are titled the molar ratio of 2-methylimidazole, zinc acetate hexahydrate, and ICG; (B) Loading efficiency and encapsulation efficiency of ICG vary with the molar ratio of 2-methylimidazole: zinc acetate hexahydrate: ICG. (n = 3 independent samples, mean ± SD).

**Figure S9**. Encapsulation efficiency and loading capacity of TPT in Z@IP NPs determined by HPLC analysis. (A) Calibration curve of TPT showing the linear relationship between concentration and HPLC peak area. (B) Encapsulation efficiency and loading capacity of TPT in Z@IP NPs calculated based on HPLC quantification. (n = 3 independent samples, mean ± SD).
**Figure S10**. High-magnification TEM photograph of Z@IP and cDZ@IP NPs (1. Locally enlarged high-resolution TEM images of Z@IP component, 2. Locally enlarged high-resolution TEM images of dopamine component).

**Figure S11**. TEM image of cDZ@IP NPs.

**Figure S12.** UV–vis absorption spectra of DZ and cDZ nanoparticles.

**Figure S13**. Quantitative analysis of ^1^O_2_ yield of cDZ@IP NPs with different environment by SOSG (n = 3 independent samples, mean ± SD).

**Figure S14**. (A) Concentration-dependent, (B) intensity-dependent, and (C) time-dependent ^1^O_2_ generation of cDZ@IP NPs under laser irradiation.

**Figure S15**. Concentration-dependent photothermal performance of cDZ@P NPs under laser irradiation.

**Figure S16**. Laser-power-dependent photothermal performance of cDZ@IP NPs under laser irradiation.

**Figure S17**. Temperature-change curves of cDZ@IP NPs with four repeated heating/cooling laser irradiation.

**Figure S18**. Quantitative analysis of cellular internalization through flow cytometry. (A) Representative flow cytometry histograms showing the intracellular fluorescence intensity of Ctrl, DZ@IP, and cDZ@IP treated cells. (B) Quantitative analysis of mean fluorescence intensity of cells after treatment with different formulations, indicating enhanced cellular uptake of cDZ@IP compared with DZ@IP. Data are presented as mean ± SD (n = 3 independent experiments).

**Figure S19**. (A) Flow cytometry and (B) quantitative analysis of intracellular ROS level after various treatment (n = 3 independent samples, mean ± SD).

**Figure S20**. Quantitative analysis of mitochondrial membrane potential by flow cytometry (n = 3 independent samples, mean ± SD).

**Figure S21**. Fluorescence images of mitochondrial membrane potential changes in MC38 cells treated with different nanoparticles (Scale bars: 100 μm).

**Figure S22**. Cell viability of MC38 cells after different treatments. (n = 4 independent samples, mean ± SD).

**Figure S23**. Flow cytometry analysis of treatment-induced cell death in MC38 cells. (A) Representative Annexin V-FITC/propidium iodide (PI) flow cytometry plots of MC38 cells after different treatments. Q4 represents viable cells (Annexin V^-^/PI^-^), Q3 represents early apoptotic cells (Annexin V^+^/PI^-^), Q2 represents late apoptotic cells (Annexin V^+^/PI^+^), and Q1 represents necrotic cells (Annexin V^-^/PI^+^). (B) Quantitative analysis of Annexin V-positive cells (early + late apoptosis) in different treatment groups. Data are presented as mean ± SD (n = 3 independent experiments).

**Figure S24**. Evaluation of the respective contributions of free TPT and nanoparticle-loaded TPT to cytotoxicity and DNA damage in MC38 cells. (A) Cell viability assessed by CCK-8 assay after different treatments. (B) Representative immunofluorescence images of γ-H2AX (green) and nuclei stained with DAPI (blue), indicating DNA damage levels. Scale bars, 20 μm. (C) Quantitative analysis of γ-H2AX fluorescence intensity (mean fluorescence intensity, MFI). Data are presented as mean ± SD (n = 3 independent experiments).

**Figure S25**. Quantitative analysis of Western blot results shown in Figure 5E. The relative expression levels of phosphorylated STING, TBK1, and IRF3 were normalized to their corresponding total protein levels (p-STING/STING, p-TBK1/TBK1, and p-IRF3/IRF3). Data are presented as mean ± SD (n = 3 independent experiments).

**Figure S26**. Immunofluorescence staining of IRF3 nuclear translocation in MC38 cells following different treatments. Representative confocal images showing IRF3 (green) and nuclei (DAPI, blue). Increased nuclear localization of IRF3 is observed after cDZ@IP + L treatment (Scale bars: 20 μm).

**Figure S27**. (A) IVIS imaging of tumor-bearing mice that received systemic administration of Cy7.5-labeled DZ@IP or cDZ@IP or free Cy7.5 (n = 3). (B) IVIS imaging of tumors and major organs from the tumor-bearing mice treated as stated above after 72 h (n = 3).

**Figure S28**. Quantitative analysis on the fluorescence intensity of the tumor sites at various time intervals (n = 3 independent samples, mean ± SD).

**Figure S29.** Circulatory half-life of cDZ@IP NPs *in vivo* (n = 3 biologically independent samples, mean ± SD).

**Figure S30.** (A) Biodistribution of cDZ@IP NPs in main organs and tumors at different time points. (B) Detection of Zn in urine after injection of cDZ@IP NPs at various time points. (C) Fluorescence intensity ratio between *ex vivo* kidney and tumor (n = 3 independent samples, mean ± SD).

**Figure S31**. Temperature variation at the tumor site in mice during treatment, monitored to assess the photothermal effect of the therapy.

**Figure S32**. Photograph of tumors after 18 days treatment in different groups.

**Figure S33**. (A)Weights, and (B) inhibitory effects in different groups (n = 5 biologically independent samples, mean ± SD).

**Figure S34**. Representative digital images of H&E staining of tumor tissues from different groups (Scale bars: 100 μm).

**Figure S35**. Body weight curves of mice after different treatments (n = 5 biologically independent samples, mean ± SD).

**Figure S36**. Representative flow cytometry gating strategies for mature DC cells (CD80^+^CD86^+^) in the tumor from Figure 6.

**Figure S37**. Flow cytometry analysis and quantification of matured DCs (**A, B**), CD4^+^ T cells (**C, D**), and CD8^+^ T cells (**E, F**) in the spleen after various treatments (n = 3 biologically independent samples, mean ± SD, statistical significance was calculated by one-way ANOVA test).

**Figure S38**. Western blot analysis of STING signaling pathway proteins in MC38 tumor tissues after different treatments. (A) Representative immunoblot images showing the expression of total STING, phosphorylated STING (p-STING), total TBK1, phosphorylated TBK1 (p-TBK1), total IRF3, and phosphorylated IRF3 (p-IRF3). (B) Quantitative densitometric analysis of protein phosphorylation levels, expressed as the ratio of phosphorylated to total protein (p-STING/STING, p-TBK1/TBK1, and p-IRF3/IRF3). Data are presented as mean ± SD (n = 3 independent experiments).

**Figure S39**. Evaluation of the content of cGAMP in tumor tissues after different treatments. (n = 3 biologically independent samples, mean ± SD, statistical significance was calculated by one-way ANOVA test).

**Figure S40**. Representative flow cytometry gating strategies for CD4^+^ T cells and CD8^+^ T cells in the tumor from Figure 6.

**Figure S41**. Representative immunofluorescence imaging of CD8^+^ T cells in tumor tissues after different treatments (Scale bars: 40 μm).

**Figure S42**. Histological changes of major organs after 18 days of treatments were evaluated by typical H&E staining (Scale bar: 200 μm).

**Figure S43**. The biochemical blood analysis (A) and normal hematology parameters (B) of MC38 tumor-bearing mice after 18 days of different treatments.

**Figure S44**. Hemolysis analysis of cDZ@IP at various concentrations. DI water and PBS were used as positive and negative controls, respectively. Representative images are presented. (n = 3 biologically independent samples, mean ± SD).

**Figure S45**. Heatmap showing the differential gene expression between the cDZ@IP + L and control groups.

**Figure S46**. Representative flow cytometry gating strategies for mature DC cells (CD80^+^CD86^+^) in the tumor from Figure 9.

**Figure S47**. Representative flow cytometry gating strategies for CD4^+^ T cells and CD8^+^ T cells in the tumor from Figure 9.

**Figure S48**. Representative flow cytometry gating strategies for GZB^+^CD8^+^, IFN-γ^+^CD8^+^, and Perforin⁺ CD8⁺ T cells in the tumor from Figure S42.

**Figure S49**. Flow cytometric analysis of GZB^+^CD8^+^ (A), IFN-γ^+^CD8^+^ (B), and Perforin⁺ CD8⁺ (C) T cells in tumor.

**Figure S50**. Representative flow cytometry gating strategies for Tcms (CD4^+^/CD8^+^CD62L^+^CD44^+^) and Tems (CD4^+^/CD8^+^CD62L⁻CD44^+^) in tumor from Figure 9.

**Figure S51**. Flow cytometry analysis (**A**) and quantification (**B**) of the ratios of and CD4⁺ T memory subsets (Tcms: CD62L⁺CD44⁺; Tems: CD62L⁻CD44⁺) in tumors (n = 3 biologically independent samples, one-way analysis of variance with Tukey’s test).

**Supplementary Table 1.** The antibodies used for Western blotting, Immunofluorescence staining and Flow cytometry.

| Antibodies | Source | Cat (Clone) | Dilutions |
| --- | --- | --- | --- |
| CRT Antibody | Cell Signaling Technology | 12238 | 1:500 |
| HMGB1 Antibody | Abcam | Ab79823 | 1:250 |
| CD8a | Proteintech | 29896-1-AP | 1:250 |
| STING | Cell Signaling Technology | 50494T | 1:1000 |
| Phospho-STING | Cell Signaling Technology | 72971T | 1:1000 |
| TBK1/NAK | Cell Signaling Technology | 3504T | 1:1000 |
| Phospho-TBK1/NAK | Cell Signaling Technology | 5483T | 1:1000 |
| IRF-3 | Cell Signaling Technology | 4302T | 1:1000 |
| Phospho-IRF-3 | Cell Signaling Technology | 29047T | 1:1000 |
| GAPDH | Cell Signaling Technology | 5174T | 1:1000 |
| BV510 Live/Dead | BD bioscience | 564406 | 0.2μg/100μL |
| Rat Anti-Mouse CD16/CD32 | BD bioscience | 553141 | 0.2μg/100μL |
| APC/Cy7 Anti-Mouse CD45 | BD bioscience | 557659 | 0.2μg/100μL |
| BV421 Anti-Mouse CD4 | BD bioscience | 740007 | 0.2μg/100μL |
| R718 Anti-Mouse CD8 | BD bioscience | 566985 | 0.2μg/100μL |
| PE/Cy7 Anti-Mouse CD44 | BD bioscience | 560569 | 0.2μg/100μL |
| PerCP-Cy5.5 Anti-Mouse CD62L | BD bioscience | 560513 | 0.2μg/100μL |
| AF647 Anti-Mouse IFN-γ | BD bioscience | 557735 | 0.2μg/100μL |
| PerCP-eF710 Anti-Mouse Granzyme B | eBioscience | 46-8898-82 | 0.2μg/100μL |
| PE Anti-Mouse Perforin | eBioscience | 12-9392-82 | 0.2μg/100μL |
| BV605 Anti-Mouse CD3 | Biolegend | 100351 | 0.2μg/100μL |
| PE Anti-Mouse CD11c | Biolegend | 117307 | 0.2μg/100μL |
| FITC Anti-Mouse CD80 | Biolegend | 104706 | 0.2μg/100μL |
| APC Anti-Mouse CD86 | Biolegend | 105011 | 0.2μg/100μL |
| Goat Anti-Rabbit IgG (H+L)  (Alexa Fluor® 488) | Abcam | ab150077 | 1:400 |
| Goat Anti-Rabbit IgG (H+L)  (Alexa Fluor® 594) | Abcam | ab150080 | 1:400 |
| Goat Anti-Rabbit IgG H&L  (HRP) | Cell Signaling Technology | 7074P2 | 1:5000 |
